# Synthesis of Novel Nitrogen-Containing Heterocycle Bromophenols and Their Interaction with Keap1 Protein by Molecular Docking

**DOI:** 10.3390/molecules22122142

**Published:** 2017-12-04

**Authors:** Xiu E. Feng, Qin Jin Wang, Jie Gao, Shu Rong Ban, Qing Shan Li

**Affiliations:** 1 School of Pharmaceutical Science, Shanxi Medical University, 56 Xinjian South Road, Taiyuan 030001, China; xiuefeng@163.com (X.E.F.); qinjinwang90@163.com (Q.J.W.); jiegao1989@163.com (J.G.); shurongban@163.com (S.R.B.); 2 Shanxi Key laboratory of Chronic Inflammatory Targeted Drugs, School of Chinese Materia Medica, Shanxi University of Traditional Chinese Medicine, 121 University Street, Jinzhong 030619, China

**Keywords:** heterocycle, bromophenol, synthesis, molecular docking, kelch-like ECH-associated protein 1 protein

## Abstract

We previously reported 5,2’-dibromo-2,4’,5’-trihydroxydiphenylmethanoe (**LM49**), a bromophenol analogue that shows strong protection from oxidative stress injury owing to its superior anti-inflammatory, antioxidant, and anti-apoptotic properties. A series of novel nitrogen-containing heterocycle bromophenols were herein synthesized by introducing substituted piperidine, piperazine, and imidazole to modify 2-position of the lead compound **LM49**. By further evaluating their cytoprotective activity against H_2_O_2_ induced injury in EA.hy926 cells, 14 target bromophenols showed moderate-to-potent activity with EC_50_ values in the range of 0.9–6.3 μM, which were stronger than that of quercetin (EC_50_: 18.0 μM), a positive reference compound. Of these, the most potent compound **22b** is a piperazine bromophenol with an EC_50_ value of 0.9 μM equivalent to the **LM49**. Molecular docking studies were subsequently performed to deduce the affinity and binding mode of derived halophenols toward the Keap1 Kelch domain, the docking results exhibited that the small molecule **22b** is well accommodated by the bound region of Keap1-Kelch and Nrf2 through stable hydrogen bonds and hydrophobic interaction, which contributed to the enhancement of affinity and stability between the ligand and receptor. The above facts suggest that **22b** is a promising pharmacological candidate for further cardiovascular drug development. Moreover, the targeting Keap1-Nrf2 protein-protein interaction may be an emerging strategy for halophenols to selectively and effectively activate Nrf2 triggering downstream protective genes defending against injury.

## 1. Introduction

**Figure 1:**
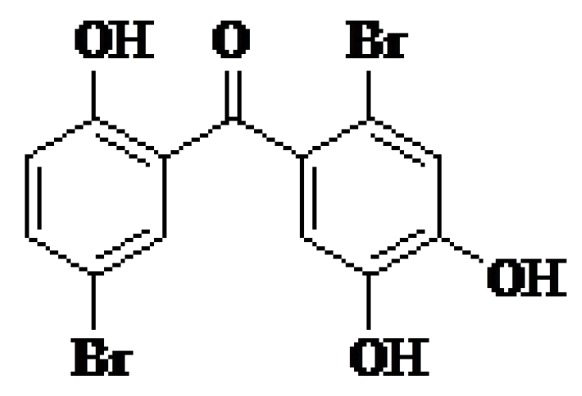
Chemical structure of the lead compound **LM49**.

The vascular endothelium is the major barrier for a cardiovascular system fighting against oxidative stress injury and inflammation [1]. In recent years, various novel skeleton halophenols derived from natural marine algaes and their derivatives obtained by structural optimization have been discovered showing the excellent vascular endothelial protective properties [2,3,4,5,6,7,8,9,10,11,12]. In support of these growing interests, we expanded upon the continuing structural optimization and mechanistic investigation on halophenols for finding a candidate compound. As a fact, we have reported the plentiful synthesis of a series of diphenylketone, diphenylmethane and phenyl furan-2-yl ketone halophenols, and their protective activity against H_2_O_2_ induced injury in human umbilical vein endothelial cells (HUVECs) [11,13]. Moreover, we indeed found a “hit” compound, 5,2’-dibromo-2,4’,5’-trihydroxydiphenylmethanoe (**LM49**) (Figure 1) possessing an EC_50_ value of 0.4 μM with the strong vascular endothelium protective ability [11], as evidenced being attributed to its anti-apoptotic, antioxidant, and anti-inflammatory abilities by further mechanical study [1,14]. To our knowledge, nitrogen-containing heterocycle derivatives have been reported to exist in many natural products and applied in many fields such as medicines and chemical products [15]. The existence of a heterocyclic unit in numerous natural products often plays an essential role in their biological activities [16,17,18,19,20,21,22]. Moreover, it helps the dosage form design of drugs such as those being prepared for injection. Based on these considerations, we focused on introducing substituted piperidine, piperazine and imidazole to modify the 2-position of lead compound **LM49** to synthesize a series of analogues aiming to find the promising pharmacological candidates for further cardiovascular drug development.

In addition, sustained oxidative stress and elevated redox state are the major causes of the development of chronic inflammation related cardiovascular diseases such as atherosclerosis and diabetes. The Keap1 (Kelch-like ECH-associated protein 1)-Nrf2 (nuclear factor erythroid 2-related factor 2)-ARE pathway plays a key role in the endogenous antioxidant system. Under basal conditions, the antioxidant transcription factor Nrf2 is bound to Keap1 protein and targets proteasomal degradation in the cytoplasm. In response to cellular injury, Nrf2 dissociates from Keap1 and activates the transcription of protective genes, defending against injury [1,23]. In our recent study, we reported that diphenylketone halophenols can protect vascular endothelial cells against the oxidative stress injury and inflammation by the activation of Nrf2 up-regulating heme oxygenase-1 (HO-1) protein expression [1], which prompted us to investigate the influence of halophenols on the Keap1-Nrf2 protein-protein interaction (PPI). Inspired by the above, we herein investigated the action mode and mechanism of halophenols interacting with the Keap1 by molecular docking.

## 2. Results and Discussion

### 2.1. Chemistry

**Scheme 1:**
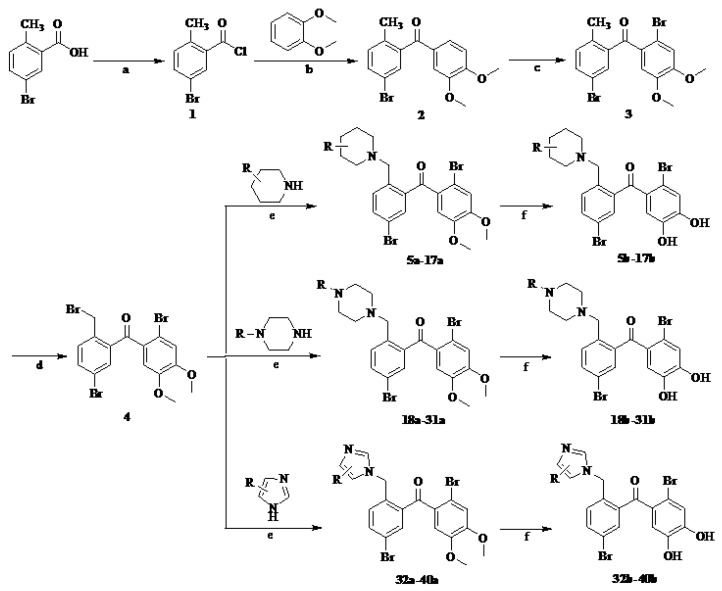
Synthetic route of target compounds. Reagents and conditions: (a) SOCl_2_ (16.1 eq), *N*,*N*-dimethyl formamide, reflux, 7 h, 70%; (b) CH_2_Cl_2_, AlCl_3_, r.t., 3 h, 90%; (c) CH_3_COOH, CH_2_Cl_2_, Br_2_ (7.1 eq), r.t., 0.5 h, 77%; (d) *N*-bromosuccinimide (1.05 eq), benzoyl peroxide (0.1 eq), CH_2_Cl_2_, sun light, r.t., 5 h, 60%; (e) CH_2_Cl_2_, anhydrous Na_2_CO_3_ (1.0 eq), r.t., 12 h, 70–93%; (f) CH_2_Cl_2_, BBr_3_ (3.1 eq), −78 °C to r.t, 1.5–2.5 h, 38–85%.

In this paper, 36 new target bromophenols were prepared by Friedel-Crafts acylation, aromatic bromination, radical substitution, nitrogen-containing heterocyclic nucleophilic substitution, and demethylation reaction according to the preparation route described in Scheme 1. All structures of target compounds were confirmed by ESI-MS, ^1^H-NMR, and ^13^C-NMR spectrum. The obtained active bromophrnols were further characterized by IR and HR-MS spectra.

The intermediate **1** was prepared from 5-bromo-2-methyl benzoic acid with anhydrous SOCl_2_ dropped little *N*,*N*-dimethyl formamide (DMF) via acylating chlorination, then reacted with 1,2-dimethoxybenzene catalyzed by AlCl_3_ to yield intermediate **2**. Aluminum chloride is an effective and cheap Lewis acid catalyst and is widely used in Friedel-Crafts acylation. Bromination reaction of intermediate **2** was quickly conducted with bromine to obtain the important compound **3** in acetic acid solvent at room temperature by electrophilic substitution in benzene ring, and was subsequently reacted with *N*-bromosuccinimide (NBS) to gain the key intermediate **4** in anhydrous CH_2_Cl_2_ using benzoyl peroxide (BPO) as the catalyst by free radical substitution. In this process, sunlight was beneficial to accelerate reaction velocity and shorten reaction time [24]. Compound **4** was treated with substituted piperidine, piperazine or imidazole in the presence of anhydrous Na_2_CO_3_ to prepare important intermediates **5a**–**40a**. Then, **5a**–**40a** were demethylated with BBr_3_ as the demethylation reagent in anhydrous CH_2_Cl_2_ at −78 °C to obtain target bromophenols **5b**–**40b** in moderate to high yields.

### 2.2. Biological Evaluation

To assess the cytoprotective activity of all synthesized target compounds **5b**–**40b** compared to important intermediates **5a**–**40a** against H_2_O_2_ induced injury in endothelial-derived EA.hy926 cells by MTT assay, we first conducted the preliminary screening to test their cytoprotective rates at a concentration of 10 μM. If the protective rates of tested compounds were higher than 45% then their EC_50_ (50% effective concentration) values were determined by examining cell viability at different concentrations of 0.3125, 0.625, 1.25, 2.5, 5, 10 μM, as presented in Table 1, Table 2 and Table 3, the values are the average of at least three independent experiments. Quercetin was used as a positive reference standard. The activity data showed that 14 target bromophenols **11b**–**14b**, **16b**, **21b**, **22b**, **24b**–**26b**, **35b**–**38b** and 15 key intermediates **5a**, **10a**, **14a**, **15a**, **17a**, **21a**, **24a**, **27a**–**32a**, **39a**, **40a** exhibited moderate-to-potent activity with EC_50_ values in the range of 0.9–7.4 μM, which were stronger than that of quercetin (EC_50_: 18.0 μM). The most promising bromophenol derivative **22b** showed the highest activity with an EC_50_ value of 0.9 μM, which was almost identical to that of the lead compound **LM49** (EC_50_: 0.7 μM). Due to the presence of a piperazine ring, compound **22b** suggests the preferably potential druggability in comparison with **LM49**.

### 2.3. Structure-Activity Relationships

Based on the data of cytoprotective activity listed in the Table 1, Table 2 and Table 3, the preliminary structure-activity relationships (SARs) of novel bromophenols analogues could be summarized. Target compound **11b**–**13b**, **16b**, **21b**, **22b**, **24b**–**26b**, **35b**–**38b** with two hydroxyl groups displayed better activity than their corresponding intermediates **11a**–**13a**, **16a**, **21a**, **22a**, **24a**–**26a**, **35a**–**38a**. These findings revealed that the presence of hetercycles and hydroxyl groups contributes to the increase of their anti-oxidative stress abilities, which is consistent with our previously presented results [11].

In 26 piperidine analogues (Table 1), five bromophenol derivatves **11b**–**14b**, **16b** with EC_50_ values of 1.4–6.1 μM and five intermediates **5a**, **10a**, **14a**, **15a**, **17a** with EC_50_ values of 1.7–5.2 μM exhibited moderate-excellent activity. Bromophenol derivatives **5b**–**8b** with no substituted groups or only a single methyl group that existed in the *ortho*-, *meta*- or *para*-position of piperidine, showed no activity. Two isomers **13b** and **9b** with two methyl groups in the *ortho*- or *meta*-position of nitrogen atom, respectively, displayed significantly different activity, bromophenol **13b** demonstrated higher activity with an EC_50_ value 5.4 μM than compound **9b**. Compound **14b** with an EC_50_ value 6.1 μM, all hydrogen atoms in the *ortho*-position of nitrogen atom replaced b**y** methyl group, exhibited nearly the same activity to compound **13b**, moreover, corresponding intermediate **14a** possessed more potent activity with an EC_50_ value 1.9 μM than bromophenol derivative **14b**, which indicated that the presence of methyl groups in two *ortho*-positions of nitrogen atom favored for the activity. In addition, compounds **15b** and **17b** are isomers with a hydroxymethyl group in the *ortho*- or *para*-position of nitrogen atom, no activity was observed. However, their corresponding intermediates **15a** and **17a** showed better activity with an EC_50_ value of 1.7 μM and 5.2 μM, respectively. Bromophenol derivative **11b**, the *meta*-position of nitrogen atom replaced by withdrawing group ethoxycarbonyl, demonstrated excellent activity with an EC_50_ value 1.4 μM compared with compound **7b** and **9b** substituted by donating group methyl**.**

Among 28 piperazine analogues (Table 2), five bromophenol derivatives **21b**, **22b**, **24b**–**26b** showed moderate-potent activity with EC_50_ values in the range of 0.9–6.3 μM, seven key intermediates **21a**, **24a**, **27a**–**31a** exhibited middle activity with EC_50_ values of 2.2–7.4 μM. Target compound **22b** showed the most potent protective activity with an EC_50_ value 0.9 μM, which was comparable to the lead compound **LM49** (EC_50_ = 0.7 μM). Replacement of 4-position of piperazine by methyl, isopropyl or diphenylmethyl group, bromophenol derivatives **24b**–**26b** displayed moderate-superior activity with EC_50_ values of 6.3, 2.2 and 1.5 μM, respectively. To bromophenol derivatives **18b**, **20b**, **23b**, and **29b**–**30b**, 4-position hydrogen of piperazine was replaced by acyl-, nitro- or fluro-substituted phenyl group, their activity was disappeared. Conversely, compound **21b**, with a methoxyl group on the 2-position of piperazine, showed better activity. Evidently, the electron withdrawing effect on the benzene ring exerted a negative effect on the activity. The above results suggest that the electronic effect and steric hindrance effect at the 4-position of piperazine play a pivotal role to the cytoprotective activity of bromophenols.

In 18 prepared imidazole analogues (Table 3), three intermediates **32a**, **39a** and **40a** showed moderate activity with EC_50_ values of 1.9 μM, 4.0 μM, and 3.2 μM, respectively. Target bromophenol derivatives **35b**–**38b**, replaced by ethyl, isopropyl or phenyl group on the 2-position of imidazole, showed excellent activity with EC_50_ values of 1.3–1.6 μM. For bromophenol derivatives **32b**, **33b**, **34b** and **39b**, with no substituent or one to two methyl groups on the imidazole, no activity was observed. From these, we can conclude that the substitution groups such as ethyl, isopropyl and phenyl existed in the 2-position of imidazole and contributed to the activity improvement. Clearly, the protective activity of imidazole bromophenols is ascribed to the electron donating effect of alkyl groups.

### 2.4. Molecular Docking Study

Nrf2 contains multiple basic residues and possesses a tight four-residue β-hairpin conformation comprising of the residues Asp-77, Glu-78, Glu-79, Thr-80, Gly-81. In particular, Glu-79 is one of the critical functional residues in the interaction of Keap1 protein and Nrf2, the side chain of which is wedged between Arg-415 and Arg-508. The unique feature of Arg-415, adopting an unusual left-handed helical conformation (58°, 49°), may cause the potential interaction of Arg-415 with Glu-79. When the ligand occupies the region closed to Arg-415, this may result in the change of the rotational isomer of Arg-415 and may also affect the electrostatic interaction. When the ligand enters the bound region of Keap1-Kelch and Nrf2, it may influence the nature of the residue Arg-415 in the active site, causing a series of changes in the electrostatic force and the acting force to weaken the interaction with Glu-79, and then bringing the dissociation of Nrf2 into the nucleus, completing the task of protein expression [23,24,25,26,27].

**Figure 2:**
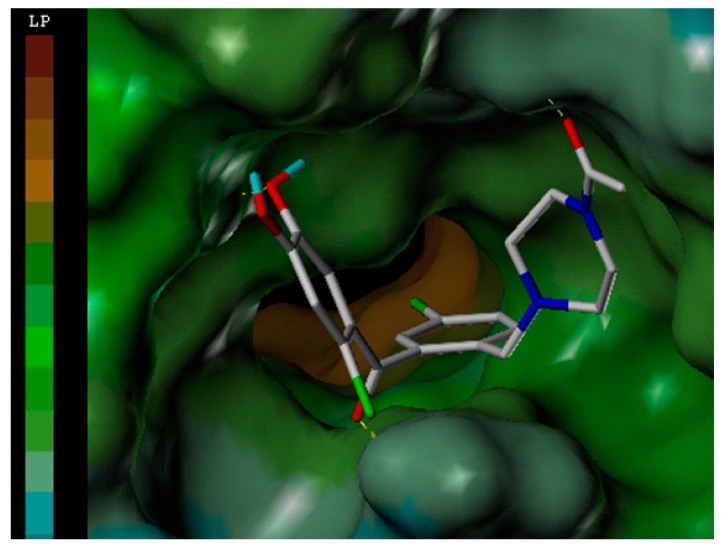
The potential energy diagram of compound **22b** binding with the Keap1 Kelch domain. (The small molecule **22b** was presented by a stick model.)

**Figure 3:**
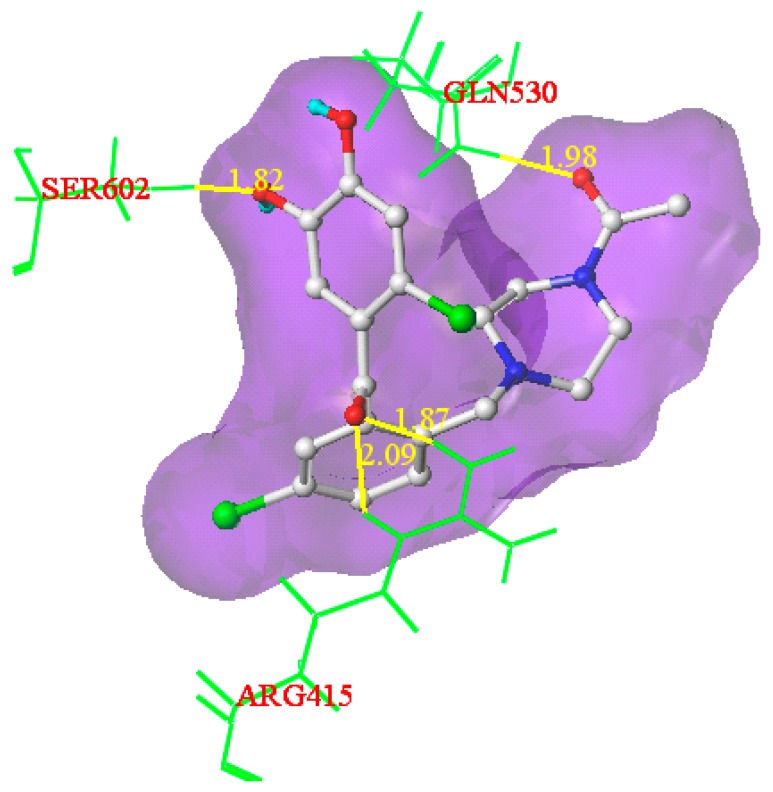
The binding modes of compound **22b** with Keap1 Kelch domain showing interacting amino acids and H-bonds. (Green shows the interacting amino acid residues, yellow dotted lines show the formed hydrogen bonds.)

In the current study, the most potent compound **22b** was employed to investigate the binding modes of derived halophenols to the kelch domain of keap1 protein by molecular docking experiment. As can be seen from the left side of Figure 2, the closer to the brown it was, the higher lipotropy or hydrophobicity it showed. Conversely, the nearer to the blue it was, the stronger hydrophily it exhibited. The cavity of the receptor presented with a brown color, which suggested strong hydrophobicity. The benzene ring, a hydrophobic group, approached the inside of the cavity. In parallel, the hydrophile groups hydroxyls and carbonyls closed to the hydrophile area of the receptor. The docking results (Figure 2) showed that a small molecule **22b** was well accommodated in the active pocket of the receptor and entered the bound region of Keap1-Kelch and Nrf2, and also exhibited excellent interaction via hydrogen bonds and hydrophobic interaction. Figure 3 showed that the amino acids in the distance of 5A from small molecule included Ser-602, Arg-415 and Gln-530. The hydroxyl group of **22b** was 1.82A distant from the Ser-602 residue. The carbonyl group linked to the two benzene rings was 1.87A and 2.09A away from Arg-415, respectively. The distance between the carbonyl group on the piperazine ring and the Gln-530 was 1.98A. The presence of multiple hydrogen bonds together with hydrophobic interaction contributed to the enhancement of affinity and stability between the ligand and receptor.

## 3. Experimental Section

### 3.1. Chemistry

The main reagents including 5-bromo-2-methyl benzoic acid, dimethoxybenzene, substituted piperdine, piperazine and imidazole were purchased from *J & K* Chemical Technology. Other chemical reagents and solvents were commercially available unless otherwise indicated. Dichloromethane was distilled from calcium hydride.

Melting points were taken on a micromelting point apparatus, which were uncorrected. The IR spectra of the compounds were recorded using a Thermo Scientific Nicolet iS 50 Fourier transform IR (FTIR) spectrometer. The ^1^H- and ^13^C-NMR spectra were recorded with a Bruker-AV 600 spectrometer in CDCl_3_ or DMSO-*d*_6_ with TMS as reference. Chemical shifts (*δ* values) and coupling constants (*J* values) were given in ppm and Hz, respectively. ESI mass spectra were obtained on an API QTRAP 3200 MS spectrometer, and HR-MS were recorded on a Bruker Daltonics Apex IV 70e FTICR-MS (Varian 7.0T).

#### 3.1.1. Preparation of Key Intermediate Compound **4**

5-Bromo-2-methyl benzoic acid (4.5 g, 7.0 mmol) was dissolved in 24 mL dried SOCl_2_ with a few drops DMF, the mixture was refluxed for 7 h. The solvent was evaporated under reduced pressure to give compound **1** as a transparent liquid. Dimethoxybenzene 4.5 mL (35.4 mmol) was added to 30 mL dried CH_2_Cl_2_ and stirred at 0 °C. Next, anhydrous AlCl_3_ (3.0 g, 22.7 mmol) was added portion-wise. The obtained compound **1** was then added to the solution, which was allowed to warm to room temperature and stirred for 3 h and quenched with 30 mL distilled water. The organic phase was separated, washed with 30 mL water and dried over anhydrous Na_2_SO_4_, and then concentrated via rotary evaporation. The crude product was purified by silica gel chromatography with ethyl acetate–petroleum ether (*v*/*v*, 1/8) as the eluent to afford compound **2**. The product was recrystallized from methanol to give a white powder in 63% total yield. m.p. 102.0–104.0 °C; ^1^H-NMR (600 MHz, DMSO-*d*_6_) *δ*: 2.53 (s, 3H, Ar-2-CH_3_), 3.86 (s, 3H, Ar-5’-OCH_3_), 3.94 (s, 3H, Ar-4’-OCH_3_), 6.99 (s, 1H, Ar-6’-H), 7.05 (s, 1H, Ar-6-H), 7.20 (t, *J* = 10.4 Hz,1H, Ar-3’-H), 7.31(d, *J* = 11.4 Hz, 1H, Ar-3-H), 7.34 (d, *J* = 11.4 Hz, 1H, Ar-4-H), 7.41 (t, *J* = 10.4 Hz, 1H, Ar-2’-H); ESI-MS *m*/*z* (%): 334.88, 336.93 ([M + H]^+^, 100, 98).

Compound **2** 2.3 g (5.5 mmol) was dissolved in the mixed solvent of 30 mL acetic acid and 8 mL dichloromethane. Next the bromine 2 mL was added to the mixture. The reaction process was monitored by thin layer chromatography (TLC). After being stirred for 0.5 h at room temperature, the mixture was slowly poured into 50 mL strong ammonia water and then cooled to room temperature. The mixture was extracted twice with CH_2_Cl_2_ (2 × 30 mL). The combined organics were washed to neutral with water, dried over anhydrous Na_2_SO_4_, and then concentrated via rotary evaporation. The crude product was purified by silica gel chromatography with ethyl acetate–petroleum ether (*v*/*v*, 1/16) as the eluent to gain 2.19 g white power compound **3** in 77% yield. m.p. 105.0–107.0 °C; ^1^H-NMR (600 MHz, DMSO-*d*_6_) *δ*: 2.64 (s, 3H, Ar-2-CH_3_), 3.87 (s, 3H, Ar-5’-OCH_3_), 3.92 (s, 3H, Ar-4’-OCH_3_), 6.93 (s, 1H, Ar-6’-H), 7.20 (t, *J* = 11.4 Hz, 1H, Ar-3-H), 7.32 (s, 1H, Ar-3’-H), 7.34 (s, 1H, Ar-6-H), 7.44 (t, *J* = 11.4 Hz, 1H, Ar-4-H). ESI-MS *m*/*z* (%): 412.85, 414.91, 416.81 ([M + H]^+^, 51, 100, 49).

Compound **3** 1.0 g (2.4 mmol), NBS 0.45 g (2.5 mmol) and BPO 58 mg (0.24 mmol) was added to 5 mL dried CH_2_Cl_2_, the mixture was stirred for 5 h in sunlight. The solvent was evaporated via rotary evaporation. The crude product was purified by silica gel chromatography with ethyl acetate–petroleum ether (*v*/*v*, 1/16) as the eluent to obtain 0.71 g pale yellow solid compound **4** in 60% yield. m.p. 103.3–105.0 °C; ^1^H-NMR (600 MHz, DMSO-*d*_6_) *δ*: 3.87 (s, 3H, Ar-5’-OCH_3_), 3.96 (s, 3H, Ar-4’-OCH_3_), 4.76 (s, 2H, Ar-2-CH_2_-), 7.04 (s, 1H, Ar-3’-H), 7.08 (s, 1H, Ar-6’-H), 7.44 (d, *J* = 12.0 Hz, 1H, Ar-3-H), 7.46 (d, *J* = 3.0 Hz, 1H, Ar-6-H), 7.64 (dd, *J* = 12.0 Hz, 3.0 Hz, 1H, Ar-4-H); ESI-MS *m*/*z* (%): 490.81, 492.81, 494.72 ([M + H]^+^, 34, 98, 100).

#### 3.1.2. General Procedure for the Synthesis of Intermediate Compounds **5a**–**40a**

Compound **4** 0.2 g (0.41 mmol) and 25 μL piperdine (0.82 mmol) was added to the 1.0 mL dried CH_2_Cl_2_. Anhydrous Na_2_CO_3_ 20 mg was then added to the mixture, which was stirred for 12 h. The mixture was washed with the distilled water, the organic phase was separated and dried over anhydrous Na_2_SO_4_, and then concentrated viarotary evaporation. The crude product was purified by silica gel chromatography with petroleum ether–acetone–strong ammonia water (*v*/*v*/*v*, 8/1/0.1) as the eluent to gain 0.18 g yellow solid compound **5a** in 90% yield.

Compounds **6a**–**40a** were also obtained from intermediate **4** in a similar manner as for the preparation of **5a** in 70–93% yield. Note that the preparation of compound **13a** and **14a** was requested for the circumstance of heating and refluxing.

**Compound 5a:** Yellow solid, yield 90%, m.p. 103.3–105.0 °C; ^1^H-NMR (600 MHz, CDCl_3_) *δ*: 1.34–1.41 (m, 6H, piperidine-3’’,4’’,5’’-H), 2.16 (s, 4H, piperidine-2’’, 6’’-H), 3.37 (s, 2H, Ar-2-CH_2_-), 3.86 (s, 3H, Ar-4’-OCH_3_), 3.95 (s, 3H, Ar-5’-OCH_3_), 7.05 (s, 1H, Ar-3’-H), 7.15 (s, 1H, Ar-6’-H), 7.29 (s, 1H, Ar-6-H), 7.47 (d, *J* = 3.0 Hz, 1H, Ar-3-H), 7.51 (dd, *J* = 3.0, 12.0 Hz, 1H, Ar-4-H); ESI-MS *m*/*z* (%) 496.02, 498.08, 499.86 ([M + H]^+^, 78, 100, 98).

**Compound 6a:** Yellow solid, yield 85%, m.p. 116.0–117.3 °C; ^1^H-NMR (600 MHz, CDCl_3_) *δ*: 0.89 (d, *J* = 9.6 Hz, 3H, piperidine-2’’-CH_3_), 1.36–1.95 (m, 6H, piperidine-3’’,4’’,5’’-H), 2.30–2.56 (m, 2H, piperidine-6’’-H), 3.26 (d, *J* = 9.6 Hz, 1H, piperidine-2’’-H), 3.83 (s, 3H, Ar-4’-OCH_3_), 3.91 (s, 1H, Ar-2-CH_2_-), 3.95 (s, 3H, Ar-5’-OCH_3_), 7.08 (s, 1H, Ar-3’-H), 7.11 (s, 1H, Ar-6’-H), 7.40 (d, *J* = 3.0 Hz, 1H, Ar-3-H), 7.43 (s, 1H, Ar-6-H), 7.52 (dd, *J* = 3.0, 12.6 Hz, 1H, Ar-4-H); ESI-MS *m*/*z* (%) 510.03, 512.07, 513.85 ([M + H]^+^, 70, 100, 90).

**Compound 7a:** Yellow solid, yield 87%, m.p. 102.2–103.5 °C; ^1^H-NMR (600 MHz, CDCl_3_) *δ*:0.74 (d, *J* = 9.0 Hz, 3H, piperidine-3’’-CH_3_), 0.90 (m, 1H, piperidine-H), 1.35–1.78 (m, 6H, piperidine-H), 2.45 (m, 2H, piperidine-6’’-H), 3.38 (s, 2H, Ar-2-CH_2_-), 3.85 (s, 3H, Ar-4’-OCH_3_), 3.95 (s, 3H, Ar-5’-OCH_3_), 7.05 (s, 1H, Ar-3’-H), 7.14 (s, 1H, Ar-6’-H), 7.28 (s, 1H, Ar-6-H), 7.47 (d, *J* = 3.0 Hz, 1H, Ar-6-H), 7.53 (dd, *J* = 3.0, 12.6 Hz, 1H, Ar-4-H); ESI-MS *m*/*z* (%) 510.06, 512.09, 513.86 ([M + H]^+^, 77, 87, 100).

**Compound 8a:** Yellow solid, yield 87%, m.p. 76.2–78.0 °C; ^1^H-NMR (600 MHz, CDCl_3_) *δ*: 0.83 (d, *J* = 9.6 Hz, 3H, piperidine-4’’-CH_3_), 0.88 (d, *J* = 9.6 Hz, 1H, piperidine-4’’-H), 1.04–2.52 (m, 8H, piperidine-H,), 3.38 (s, 2H, Ar-2-CH_2_-), 3.85 (s, 3H, Ar-4’-OCH_3_), 3.94 (s, 3H, Ar-5’-OCH_3_), 7.05 (s, 1H, Ar-3’-H), 7.15 (s, 1H, Ar-6’-H), 7.29 (s, 1H, Ar-6-H), 7.47 (d, *J* = 3.0 Hz, 1H, Ar-6-H), 7.53 (dd, *J* = 3.0, 12.6 Hz, 1H, Ar-4-H); ESI-MS *m*/*z* (%) 510.03, 512.04, 513.90 ([M + H]^+^, 55, 100, 65).

**Compound 9a:** Yellow solid, yield 86%, m.p. 82.2–84.0 °C; ^1^H-NMR (600 MHz, CDCl_3_) *δ*: 0.72 (s, *3*H, piperidine-3’’-CH_3_), 0.74 (s, 3H, piperidine-5’’-CH_3_), 0.86–2.46 (m, 8H, piperidine-H), 3.38 (s, 2H, Ar-2-CH_2_-), 3.84 (s, 3H, Ar-4’-OCH_3_), 3.94 (s, 3H, Ar-5’-OCH_3_), 7.05 (s, 1H, Ar-3’-H), 7.13 (s, 1H, Ar-6’-H), 7.26 (s, 1H, Ar-6-H), 7.47 (d, *J* = 3.0 Hz, 1H, Ar-6-H), 7.53 (dd, *J* = 3.0, 12.0 Hz, 1H, Ar-4-H); ESI-MS *m*/*z* (%) 524.08, 526.14, 527.86 ([M + H]^+^, 75, 95, 100).

**Compound 10a:** Yellow solid, yield 75%, m.p. 48.3–49.5 °C; ^1^H-NMR (600 MHz, CDCl_3_) *δ*: 1.59–2.33 (m, 9H, piperidine-H), 3.44 (s, 2H, Ar-2-CH_2_-), 3.87 (s, 3H, Ar-4’-OCH_3_), 3.95 (s, 3H, Ar-5’-OCH_3_), 7.05 (s, 1H, Ar-3’-H), 7.16 (s, 1H, Ar-6’-H), 7.30 (s, 1H, Ar-6-H), 7.48 (d, *J* = 3.0 Hz, 1H, Ar-3-H), 7.55 (dd, *J* = 3.0, 12.0 Hz, 1H, Ar-4-H), 10.2 (s, 1H, piperidine-4-COOH); ESI-MS *m*/*z* (%) 568.13, 570.15, 571.93 ([M + H]^+^, 70, 100, 92).

**Compound 11a:** Yellow solid, yield 70%, m.p. 44.8–46.2 °C; ^1^H-NMR (600 MHz, CDCl_3_) *δ*: 1.20 (t, *J* = 7.2 Hz, 3H, piperidine-3’’-COOCH_2_-CH_3_), 1.58–2.70 (m, 9H, piperidine-H)_,_ 3.49 (q, *J* = 16.8 Hz, 2H, piperidine-3’’-COOCH_2_-), 3.85 (s, 3H, Ar-4’-OCH_3_), 3.94 (s, 3H, Ar-5’-OCH_3_), 4.07 (s, 2H, Ar-2-CH_2_-), 7.06 (s, 1H, Ar-3’-H), 7.11 (s, 1H, Ar-6’-H), 7.31 (d, *J* = 7.8 Hz, 1H, Ar-3-H), 7.46 (s, 1H, Ar-6-H), 7.53 (d, *J* = 8.4 Hz, 1H, Ar-4-H); ESI-MS *m*/*z* (%) 568.22, 570.14, 572.29 ([M + H]^+^, 100, 65, 97).

**Compound 12a:** Yellow solid, yield 75%, m.p. 62.1–63.0 °C; ^1^H-NMR (600 MHz, CDCl_3_) *δ*: 1.40–2.51 (m, 9H, piperidine-H), 3.44 (s, 2H, Ar-2-CH_2_-), 3.62 (brs, 1H, piperidine-OH), 3.85 (s, 3H, Ar-4’-OCH_3_), 3.94 (s, 3H, Ar-5’-OCH_3_), 7.06 (s, 1H, Ar-3’-H), 7.15 (s, 1H, Ar-6’-H), 7.28 (d, *J* = 7.8 Hz, 1H, Ar-3-H), 7.47 (d, *J* =1.2 Hz, 1H, Ar-6-H), 7.53 (dd, *J* = 1.2, 8.4 Hz, 1H, Ar-4-H); ESI-MS *m*/*z* (%) 512.20, 513.56, 516.26 ([M + H]^+^, 100, 65, 97).

**Compound 13a:** Yellow solid, yield 75%, m.p. 148.8–150.2 °C; ^1^H-NMR (600 MHz, CDCl_3_) *δ*: 0.92 (d, *J* = 6.0 Hz, 6H, piperidine-2’’,6’’-CH_3_), 1.29–1.58 (m, 6H, piperidine-3’’,4’’,5’’-H), 2.48 (m, 2H, piperidine-2’’,6’’-H), 3.86 (s, 3H, Ar-4’-OCH_3_), 3.89 (s, 2H, Ar-2-CH_2_-), 3.95 (s, 3H, Ar-5’-OCH_3_), 7.00 (s, 1H, Ar-3’-H), 7.06 (s, 1H, Ar-6’-H), 7.36 (d, *J* = 1.8 Hz, 1H, Ar-6-H), 7.58 (dd, *J* = 1.8, 8.4 Hz, 1H, Ar-3-H), 8.08 (d, *J* = 8.4 Hz, 1H, Ar-4-H); ESI-MS *m*/*z* (%) 524.16, 526.19, 528.03 ([M + H]^+^, 70, 100, 80).

**Compound 14a:** Yellow solid, yield 90%, m.p. 140.0–142.0 °C; ^1^H-NMR (600 MHz, CDCl_3_) *δ*: 0.85–1.02 (m, 12H, piperidine-2’’,6’’-CH_3_), 1.53 (m, 6H, piperidine-3’’_,_4’’,5’’-H), 3.86 (s, 3H, Ar-4’-OCH_3_), 3.95 (s, 3H, Ar-5’-OCH_3_), 3.96 (s, 2H, Ar-2-CH_2_-), 7.00 (s, 1H, Ar-3’-H), 7.07 (s, 1H, Ar-6’-H), 7.36 (d, *J* = 1.8 Hz, 1H, Ar-6-H), 7.57 (dd, *J* = 1.8, 8.4 Hz, 1H, Ar-3-H), 8.10 (d, *J* = 8.4 Hz, 1H, Ar-4-H); ESI-MS *m*/*z* (%) 552.21, 554.23, 556.03 ([M + H]^+^, 55, 100, 65).

**Compound 15a:** Yellow solid, yield 70%, m.p. 138.9–140.5 °C; ^1^H-NMR (600 MHz, CDCl_3_) *δ*: 1.47–1.61 (m, 6H, piperidine-3’’,4’’,5’’-H), 2.03 (t, *J* = 9.0 Hz, 2H, piperidine-6’’-H), 2.29–2.32 (m, 1H, piperidine-2’’-H), 2.75 (brs, 1H, -OH), 3.31 (d, *J* = 7.8 Hz, 2H, piperidine-CH_2_-), 3.81 (s, 2H, Ar-2-CH_2_-), 3.83 (s, 3H, Ar-4’-OCH_3_), 3.96 (s, 3H, Ar-5’-OCH_3_), 7.06 (s, 1H, Ar-3’-H), 7.10 (s, 1H, Ar-6’-H), 7.37 (d, *J* = 8.4 Hz, 1H, Ar-3-H), 7.40 (d, *J* = 1.8 Hz, 1H, Ar-6-H), 7.55 (dd, *J* = 1.8, 8.4 Hz, 1H, Ar-4-H); ESI-MS *m*/*z* (%) 526.16, 528.23, 530.03 ([M + H]^+^, 62, 100, 80).

**Compound 16a:** Yellow solid, yield 72%, m.p. 50.5–51.9 °C; ^1^H-NMR (600 MHz, CDCl_3_) *δ*: 0.88–1.54 (m, 7H, piperidine-3’’,4’’,5’’-H, piperidine-4-CH_2_-), 1.85 (t, *J* = 10.8 Hz, 2H, piperidine-2’’-H), 2.01 (brs, 1H, -OH), 2.53 (t, *J* = 10.8 Hz, 2H, piperidine-6’’-H), 3.40 (s, 2H, Ar-2-CH_2_-), 3.64 (t, *J* = 9.6 Hz, 2H, HO-CH_2_-), 3.84 (s, 3H, Ar-4’-OCH_3_), 3.94 (s, 3H, Ar-5’-OCH_3_), 7.06 (s, 1H, Ar-3’-H), 7.13 (s, 1H, Ar-6’-H), 7.29 (d, *J* = 12.0 Hz, 1H, Ar-3-H), 7.47 (d, *J* = 1.8 Hz, 1H, Ar-6-H), 7.52 (dd, *J* = 2.4, 12.0 Hz, 1H, Ar-4-H); ESI-MS *m*/*z* (%) 540.24, 542.26, 544.13 ([M + H]^+^, 65, 100, 75).

**Compound 17a:** White solid, yield 65%, m.p. 44.2–45.8 °C; ^1^H-NMR (600 MHz, CDCl_3_) *δ*: 1.09–1.58 (m, 5H, piperidine-3’’,4’’,5’’-H), 1.87 (t, *J* = 14.4 Hz, 2H, piperidine-2’’-H), 2.05 (brs, 1H, -OH), 2.57 (t, *J* = 16.8 Hz, 2H, piperidine-6’’-H), 3.42 (d, *J* = 6.0 Hz, 2H, HO-CH_2_-), 3.43 (s, 2H, Ar-2-CH_2_-), 3.84 (s, 3H, Ar-4’-OCH_3_), 3.94 (s, 3H, Ar-5’-OCH_3_), 7.06 (s, 1H, Ar-3’-H), 7.13 (s, 1H, Ar-6’-H), 7.28 (d, *J* = 12.0 Hz, 1H, Ar-3-H), 7.47 (d, *J* = 3.0 Hz, 1H, Ar-6-H), 7.52 (dd, *J* = 3.0, 12.0 Hz, 1H, Ar-4-H); ESI-MS *m*/*z* (%) 526.24, 528.32, 530.16 ([M + H]^+^, 70, 100, 80).

**Compound 18a:** White solid, yield 60%, m.p. 200.0–201.0 °C; ^1^H-NMR (600 MHz, CDCl_3_) *δ*: 2.13–2.24 (m, 8H, piperazine-H), 3.37 (s, 4H, Ar-2-CH_2_-), 3.84 (s, 6H, Ar-4’-OCH_3_), 3.94 (s, 6H, Ar-5’-OCH_3_), 7.04 (s, 2H, Ar-3’-H), 7.11 (s, 2H, Ar-6’-H), 7.24 (d, *J* = 12.0 Hz, 2H, Ar-3-H), 7.45 (d, *J* = 3.0 Hz, 2H, Ar-6-H), 7.52 (dd, *J* = 3.0, 12.0 Hz, 2H, Ar-4-H); ESI-MS *m*/*z* (%) 909.12, 911.10, 912.95 ([M + H]^+^, 65, 100, 75).

**Compound 19a:** White solid, yield 78%, m.p. 74.3–76.0 °C; ^1^H-NMR (600 MHz, CDCl_3_) *δ*: 1.03 (t, *J* = 10.8 Hz, 3H, piperazine-4’’-CH_3_), 1.70–2.36 (m, 10H, piperazine-4’’-CH_2_, piperazine-H), 3.43 (s, 2H, Ar-2-CH_2_-), 3.86 (s, 3H, Ar-4’-OCH_3_), 3.95 (s, 3H, Ar-5’-OCH_3_), 7.05 (s, 1H, Ar-3’-H), 7.15 (s, 1H, Ar-6’-H), 7.29 (s, *J* = 12.0 Hz, 1H, Ar-3-H), 7.48 (d, *J* = 3.0 Hz, 1H, Ar-6-H), 7.53 (dd, *J* = 3.0, 12.0 Hz, 1H, Ar-4-H); ESI-MS *m*/*z* (%) 525.14, 527.20, 529.00 ([M + H]^+^, 80, 85, 100).

**Compound 20a:** Yellow solid, yield 75%, m.p. 65.8–66.9 °C; ^1^H-NMR (600 MHz, CDCl_3_) *δ*: 2.42 (t, *J* = 7.2Hz, 4H, piperazine-2’’,6’’-H), 3.05 (t, *J* = 7.2 Hz, 4H, piperazine-3’’,5’’-H), 3.52 (s, 2H, Ar-2-CH_2_-), 3.85 (s, 3H, Ar-4’-OCH_3_), 3.94 (s, 3H, Ar-5’-OCH_3_), 6.82–6.87 (m, 3H, 3H, piperazine-Ar-3’’’,4’’’,5’’’-H), 7.05 (s, 1H, Ar-3’-H), 7.16 (s, 1H, Ar-6’-H), 7.24 (d, *J* = 11.4 Hz, 2H, piperazine-Ar-2’’’,6’’’-H), 7.33 (d, *J* = 12.0 Hz, 1H, Ar-3-H), 7.50 (d, *J* = 3.0 Hz, 1H, Ar-6-H), 7.57 (dd, *J* = 3.0, 12.0 Hz, 1H, Ar-4-H); ESI-MS *m*/*z* (%) 573.10, 575.09, 576.95 ([M + H]^+^, 48, 100, 58).

**Compound 21a:** Yellow solid, yield 75%, m.p. 68.2–69.7 °C; ^1^H-NMR (600 MHz, CDCl_3_) *δ*: 2.45 (s, 4H, piperazine-3’’,5’’-H), 2.92 (s, piperazine-2’’, 4’’-H), 3.50 (s, 2H, Ar-2-CH_2_-), 3.82 (s, 3H, Ar-4’-OCH_3_), 3.84 (s, 3H, Ar-5’-OCH_3_), 3.93 (s, 3H, piperazine-Ar-2’’’-OCH_3_), 6.82 (d, *J* = 8.4 Hz, 1H, piperazine-Ar-6’’’-H), 6.85 (d, *J* = 7.8 Hz, 1H, Ar-3’’’-H, Ar-6’’’-H), 6.89 (t, *J* = 7.8 Hz, 1H, Ar-4’’’-H), 6.97 (t, *J* = 7.8 Hz, 1H, Ar-5’’’-H), 7.05 (s, 1H, Ar-3’-H), 7.18 (s, 1H, Ar-6’-H), 7.31 (s, *J* = 7.8 Hz, 1H, Ar-3-H), 7.51 (s, 1H, Ar-6-H), 7.55 (dd, *J* = 1.2, 7.8 Hz, 1H, Ar-4-H); ESI-MS *m*/*z* (%) 603.26, 604.57, 607.42 ([M + H]^+^, 100, 70, 90).

**Compound 22a:** Yellow solid, yield 68%, m.p. 64.8–66.5 °C; ^1^H-NMR (600 MHz, CDCl_3_) *δ*: 2.03 (s, 3H, piperazine-4’’-COCH_3_), 2.24 (t, *J* = 4.8 Hz, 2H, piperazine-2’’-H), 2.29 (t, *J* = 5.4 Hz, 2H, piperazine-6’’-H), 3.31 (t, *J* = 4.8 Hz, 2H, piperazine-3’’-H), 3.48 (t, 2H, *J* = 5.4 Hz, piperazine-5’’-H), 3.51 (s, 2H, Ar-2-CH_2_-), 3.85 (s, 3H, Ar-4’-OCH_3_), 3.95 (s, 3H, Ar-5’-OCH_3_), 7.07 (s, 1H, Ar-3’-H), 7.13 (s, 1H, Ar-6’-H), 7.29 (d, *J* = 8.4 Hz, 1H, Ar-3-H), 7.48 (d, *J* = 1.8 Hz,1H, Ar-6-H), 7.55 (dd, *J* = 1.8, 8.4 Hz, 1H, Ar-4-H); ESI-MS *m*/*z* (%) 539.21, 541.18, 543.14 ([M + H]^+^, 85, 50, 100).

**Compound 23a:** Yellow solid, yield 60%, m.p. 64.5–66.3 °C; ^1^H-NMR (600 MHz, CDCl_3_) *δ*: 2.44 (t, *J* = 4.8 Hz, 4H, piperazine-2’’,6’’-H), 3.30 (t, *J* = 4.8 Hz, piperazine-3’’,5’’-H), 3.56 (s, 2H, Ar-2-CH_2_-), 3.85 (s, 3H, Ar-4’-OCH_3_), 3.94 (s, 3H, Ar-5’-OCH_3_), 6.75 (d, *J* = 9.6 Hz, 2H, piperazine-Ar-2’’’,6’’’-H), 7.07 (s, 1H, Ar-3’-H), 7.15 (s, 1H, Ar-6’-H), 7.34 (d, *J* = 7.8 Hz, 1H, Ar-3-H), 7.50 (d, *J* = 1.8 Hz, 1H, Ar-6-H), 7.57 (dd, *J* = 1.8, 7.8 Hz, 1H, Ar-4-H), 8.10 (d, *J* = 9.0 Hz, 2H, piperazine-Ar-3’’’,5’’’-H); ESI-MS *m*/*z* (%) 618.23, 620.12, 622.02 ([M + H]^+^, 48, 100, 58).

**Compound 24a:** Yellow solid, yield 77%, m.p. 125.0–127.0 °C; ^1^H-NMR (600 MHz, CDCl_3_) *δ*: 2.02 (t, *J* = 8.4 Hz, 8H, piperazine-H), 2.21 (s, 3H, piperazine-4’’-CH_3_), 3.44 (s, 2H, Ar-2-CH_2_-), 3.86 (s, 3H, Ar-4’-OCH_3_), 3.95 (s, 3H, Ar-5’-OCH_3_), 7.06 (s, 1H, Ar-3’-H), 7.15 (s, 1H, Ar-6’-H), 7.28 (d, *J* = 8.4 Hz, 1H, Ar-3-H), 7.48 (s, 1H, Ar-6-H), 7.53 (d, *J* = 8.4 Hz, 1H, Ar-4-H); ESI-MS *m*/*z* (%) 511.12, 513.13, 515.00 ([M + H]^+^, 50, 100, 60).

**Compound 25a:** White solid, yield 71%, m.p. 70.0–71.5 °C; ^1^H-NMR (600 MHz, CDCl_3_) *δ*: 0.97 (d, *J* = 6.6 Hz, 6H, piperazine-4’’-CH_3_), 2.28 (s, 4H, piperazine-3’’,5’’-H), 2.37 (s, 4H, piperazine-2’’,6’’-H), 2.57 (m, 1H, piperazine-4’’-CH-), 3.42 (s, 2H, Ar-2-CH_2_-), 3.85 (s, 3H, Ar-4’-OCH_3_), 3.94 (s, 3H, Ar-5’-OCH_3_), 7.06 (s, 1H, Ar-3’-H), 7.15 (s, 1H, Ar-6’-H), 7.28 (d, *J* = 8.4 Hz, 1H, Ar-3-H), 7.48 (d, *J* = 1.8 Hz, 1H, Ar-6-H), 7.52 (dd, *J* = 1.8, 8.4 Hz, 1H, Ar-4-H); ESI-MS *m*/*z* (%) 539.02, 541.10, 543.26 ([M + H]^+^, 50, 100, 50).

**Compound 26a:** Yellow solid, yield 67%, m.p. 160.0–162.0 °C; ^1^H-NMR (600 MHz, CDCl_3_) *δ*: 2.26 (s, 8H, piperazine-H), 3.43 (s, 2H, Ar-2-CH_2_-), 3.81 (s, 3H, Ar-4’-OCH_3_), 3.93 (s, 3H, Ar-5’-OCH_3_), 4.15 (s, 1H, piperazine-4’’-CH-), 7.03 (s, 1H, Ar-3’-H), 7.11 (s, 1H, Ar-6’-H), 7.15 (t, *J* = 7.8 Hz, 2H, piperazine-CH-Ar-4’’’-H), 7.23 (t, *J* = 7.8 Hz, 4H, piperazine-CH-Ar-3’’’,5’’’-H), 7.27 (d, *J* = 7.8 Hz, 1H, Ar-3-H), 7.34 (d, *J* = 7.2 Hz, 4H, piperazine-CH-Ar-2’’’,6’’’-H), 7.45 (d, *J* = 1.8 Hz, 1H, Ar-6-H), 7.50 (dd, *J* = 1.8, 7.8 Hz, 1H, Ar-4-H); ESI-MS *m*/*z* (%) 663.26, 665.29, 667.10 ([M + H]^+^, 55, 100, 72).

**Compound 27a:** Yellow solid, yield 79%, m.p. 56.0–58.0 °C; ^1^H-NMR (600 MHz, CDCl_3_) *δ*: 1.78 (s, 1H, piperazine-4’’-OH), 2.02 (s, 8H, piperazine-H), 2.47 (t, *J* = 5.4 Hz, 2H, piperazine-4’’-CH_2_-), 3.45 (s, 2H, Ar-2-CH_2-_), 3.56 (t, *J* = 5.4 Hz, 2H, HO-CH_2_-), 3.85 (s, 3H, Ar-4’-OCH_3_), 3.95 (s, 3H, Ar-5’-OCH_3_), 5.52 (brs, 1H, -OH), 7.06 (s, 1H, Ar-3’-H), 7.14 (s, 1H, Ar-6’-H), 7.28 (d, *J* = 7.8 Hz, 1H, Ar-3-H), 7.48 (d, *J* = 1.8 Hz, 1H, Ar-6-H), 7.53 (dd, *J* = 1.8, 7.8 Hz, 1H, Ar-4-H); ESI-MS *m*/*z* (%) 541.15, 543.18, 545.03 ([M + H]^+^, 60, 100, 70).

**Compound 28a:** Yellow solid, yield 71%, m.p. 146.0–148.0 °C; ^1^H-NMR (600 MHz, CDCl_3_) *δ*: 2.34 (t, *J* = 4.8 Hz, 4H, piperazine-2’’,6’’-H), 3.52 (s, 2H, Ar-2-CH_2_-), 3.68 (t, *J* = 4.8 Hz, 4H, piperazine-3’’,5’’-H), 3.85 (s, 3H, Ar-4’-OCH_3_), 3.94 (s, 3H, Ar-5’-OCH_3_), 6.45 (t, *J* = 4.8 Hz, 1H, pyrimidine-5’’’-H), 7.07 (s, 1H, Ar-3’-H), 7.16 (s, 1H, Ar-6’-H), 7.34 (d, 1H, *J* = 8.4 Hz, Ar-3-H), 7.48 (d, *J* = 1.8 Hz, 1H, Ar-6-H), 7.55 (dd, *J* = 1.8, 8.4 Hz, 1H, Ar-4-H), 8.26 (d, *J* = 4.8 Hz, 2H, pyrimidine-4’’’,6’’’-H); ESI-MS *m*/*z* (%) 575.14, 577.12, 579.02 ([M + H]^+^, 48, 100, 58).

**Compound 29a:** Yellow solid, yield 65%, m.p. 115.2–116.1 °C; ^1^H-NMR (600 MHz, CDCl_3_) *δ*: 2.43 (t, *J* = 4.8 Hz, 4H, piperazine-2’’,6’’-H), 2.97 (t, *J* = 4.8 Hz, 4H, piperazine-3’’,5’’-H), 3.52 (s, 2H, Ar-2-CH_2_-), 3.84 (s, 3H, Ar-4’-OCH_3_), 3.94 (s, 3H, Ar-5’-OCH_3_), 6.80 (dd, *J* = 4.8, 9.0 Hz, 2H, Ar-2’’’,6’’’-H), 6.93 (t, *J* = 9.0 Hz, 2H, Ar-3’’’,5’’’-H), 7.05 (s, 1H, Ar-3’-H), 7.15 (s, 1H, Ar-6’-H), 7.33 (d, 1H, *J* = 8.4 Hz, Ar-3-H), 7.50 (d, *J* = 1.8 Hz, 1H, Ar-6-H), 7.55 (dd, *J* = 1.8, 8.4 Hz, 1H, Ar-4-H); ESI-MS *m*/*z* (%) 591.16, 593.16, 595.03 ([M + H]^+^, 48, 100, 58).

**Compound 30a:** Yellow solid, yield 70%, m.p. 46.2–48.0 °C; ^1^H-NMR (600 MHz, CDCl_3_) *δ*: 2.44 (t, *J* = 7.2 Hz, 4H, piperazine-2’’,6’’-H), 2.95 (t, *J* = 7.2 Hz, 4H, piperazine-3’’,5’’-H), 3.52 (s, 2H, Ar-2-CH_2_-), 3.84 (s, 3H, Ar-4’-OCH_3_), 3.94 (s, 3H, Ar-5’-OCH_3_), 6.84–7.03 (m, 4H, piperazine-Ar-H), 7.06 (s, 1H, Ar-3’-H), 7.16 (s, 1H, Ar-6’-H), 7.32 (d, 1H, *J* = 12.0 Hz, Ar-3-H), 7.50 (d, *J* = 3.0 Hz, 1H, Ar-6-H), 7.55 (dd, *J* = 3.0, 12.0 Hz, 1H, Ar-4-H); ESI-MS *m*/*z* (%) 591.20, 593.23, 595.10 ([M + H]^+^, 65, 100, 75).

**Compound 31a:** Yellow solid, yield 83%, m.p. 129.1–130.8 °C; ^1^H-NMR (600 MHz, CDCl_3_) *δ*: 2.42 (t, *J* = 7.2 Hz, 4H, piperazine-2’’,6’’-H), 2.94 (t, *J* = 7.2 Hz, piperazine-3’’,5’’-H), 3.51 (s, 2H, Ar-2-CH_2_-), 3.75 (s, 3H, piperazine-Ar-OCH_3_), 3.84 (s, 3H, Ar-4’-OCH_3_), 3.94 (s, 3H, Ar-5’-OCH_3_), 6.79–684 (m, 4H, piperazine-Ar-H), 7.05 (s, 1H, Ar-3’-H), 7.16 (s, 1H, Ar-6’-H), 7.30 (d, *J* = 12.0 Hz, 1H, Ar-3-H), 7.50 (d, *J* = 3.0 Hz, 1H, Ar-6-H), 7.55 (dd, *J* = 3.0, 12.0 Hz, 1H, Ar-4-H); ESI-MS *m*/*z* (%) 603.19, 605.19, 607.08 ([M + H]^+^, 48, 100, 58).

**Compound 32a:** Yellow solid, yield 85%, m.p. 150.3–152.0 °C; ^1^H-NMR (600 MHz, CDCl_3_) *δ*: 3.89 (s, 3H, Ar-4’-OCH_3_), 3.96 (s, 3H, Ar-5’-OCH_3_), 5.44 (s, 2H, Ar-2-CH_2_-), 6.90 (d, *J* = 12.6 Hz, 1H, imidazole-5’’-H), 6.93 (s, *J* = 12.6 Hz, H, imidazole-4’’-H), 6.94 (d, *J* = 12.0 Hz,1H, Ar-3-H), 7.06 (s, 1H, Ar-3’-H), 7.10 (s, 1H, Ar-6’-H)), 7.47 (d, *J* = 3.0 Hz, 1H, Ar-6-H), 7.57 (s, 1H, imidazole-2’’-H), 7.60 (dd, *J* = 3.0, 12.0 Hz, 1H, Ar-4-H); ESI-MS *m*/*z* (%) 479.03, 481.07, 482.86 ([M + H]^+^, 70, 100, 88).

**Compound 33a:** Yellow solid, yield 84%, m.p. 57.2–59.0 °C; ^1^H-NMR (600 MHz, CDCl_3_) *δ*: 2.44 (s, 3H, imidazole-2’’-CH_3_), 3.90 (s, 3H, Ar-4’-OCH_3_), 3.96 (s, 3H, Ar-5’-OCH_3_), 5.37 (s, 2H, Ar-2-CH_2_-), 6.59 (d, *J* = 8.4 Hz, 1H, imidazole-5’’-H), 6.86 (s,1H, Ar-3’-H), 6.98 (d, *J* = 7.8 Hz,1H, Ar-3-H), 7.00 (d, *J* = 7.8 Hz, 1H, imidazole-4’’-H), 7.07 (s, 1H, Ar-6’-H), 7.49 (d, *J* = 1.8 Hz, 1H, Ar-6-H), 7.57 (dd, *J* = 1.8, 8.4 Hz, 1H, Ar-4-H); ESI-MS *m*/*z* (%) 493.14, 495.17, 496.97 ([M + H]^+^, 75, 100, 95).

**Compound 34a:** Yellow solid, yield 81%, m.p. 49.5–50.0 °C; ^1^H-NMR (600 MHz, CDCl_3_) *δ*: 2.22 (s, 3H, imidazole-4’’-CH_3_), 3.88 (s, 3H, Ar-4’-OCH_3_), 3.96 (s, 3H, Ar-5’-OCH_3_), 5.35 (s, 2H, Ar-2-CH_2_-), 6.63 (s, 1H, imidazole-5’’-H), 6.89 (d, *J* = 12.0 Hz, 1H, Ar-3-H), 6.94 (s, 1H, Ar-3’-H), 7.06 (s, 1H, Ar-6’-H), 7.44 (s, 1H, imidazole-2’’-H), 7.47 (d, *J* = 10.8 Hz, 1H, Ar-6-H), 7.59 (d, *J* = 12.0 Hz, 1H, Ar-4-H); ESI-MS *m*/*z* (%) 493.12, 495.17, 496.93 ([M + H]^+^, 75, 100, 98).

**Compound 35a:** White solid, yield 85%, m.p. 167.0–168.5 °C; ^1^H-NMR (600 MHz, CDCl_3_) *δ*: 1.27 (t, *J* = 10.8 Hz, 3H, imidazole-2’’-C-CH_3_), 2.59 (q, *J* = 10.8 Hz, 2H, imidazole-2’’-CH_2_-), 3.91 (s, 3H, Ar-4’-OCH_3_), 3.97 (s, 3H, Ar-5’-OCH_3_), 5.38 (s, 2H, Ar-2-CH_2_-), 6.57 (d, *J* = 12.0 Hz, 1H, Ar-3-H), 6.85 (d, *J* = 1.8 Hz, 1H, imidazole-5’’-H), 7.00 (s, 1H, Ar-3’-H), 7.04 (d, *J* = 1.8 Hz, 1H, imidazole-4’’-H), 7.07 (s, 1H, Ar-6’-H), 7.49 (d, *J* = 3.0 Hz, 1H, Ar-6-H), 7.56 (dd, *J* = 3.0, 12.0 Hz, 1H, Ar-4-H); ESI-MS *m*/*z* (%) 507.07, 509.05, 510.97 ([M + H]^+^, 50, 100, 62).

**Compound 36a:** Yellow solid, yield 77%, m.p. 162.0–163.0 °C; ^1^H-NMR (600 MHz, CDCl_3_) *δ*: 1.25 (d, *J* = 6.6 Hz, 6H, imidazole-2’’-C-CH_3_), 2.89 (m, 1H, imidazole-2’’-CH-), 3.91 (s, 3H, Ar-4’-OCH_3_), 3.97 (s, 3H, Ar-5’-OCH_3_), 5.41 (s, 2H, Ar-2-CH_2_-), 6.57 (d, *J* = 8.4 Hz, 1H, Ar-3-H), 6.81 (d, 1H, *J* = 1.2 Hz, imidazole-5’’-H), 7.00 (s, 1H, Ar-3’-H), 7.06 (d, *J* = 1.2 Hz, 1H, imidazole-4’’-H), 7.07 (s, 1H, Ar-6’-H), 7.49 (d, *J* = 1.2 Hz, 1H, Ar-6-H), 7.56 (dd, *J* = 1.8, 8.4 Hz, 1H, Ar-4-H); ESI-MS *m*/*z* (%) 521.17, 523.05, 525.04 ([M + H]^+^, 85, 98, 100).

**Compound 37a:** Yellow solid, yield 72%, m.p. 32.8–34.5 °C; ^1^H-NMR (600 MHz, CDCl_3_) *δ*: 1.24 (s, 3H, imidazole-2’’-C-CH_3_), 2.23 (s, 3H, imidazole-4’’-CH_3_), 2.58 (q, 2H, imidazole-2’’-CH_2_-), 3.90 (s, 3H, Ar-4’-OCH_3_), 3.97 (s, 3H, Ar-5’-OCH_3_), 5.30 (s, 2H, Ar-2-CH_2_-), 6.54 (s, 1H, imidazole-5’’-H), 6.63 (d, *J* = 8.4 Hz, 1H, Ar-3-H), 6.99 (s, 1H, Ar-3’-H), 7.07 (s, 1H, Ar-6’-H), 7.48 (d, *J* = 1.8 Hz, 1H, Ar-6-H), 7.57 (dd, *J* = 1.8, 8.4 Hz, 1H, Ar-4-H); ESI-MS *m*/*z* (%) 521.18, 523.22, 525.06 ([M + H]^+^, 80, 100, 95).

**Compound 38a:** White solid, yield 70%, m.p. 183.0–185.0 °C; ^1^H-NMR (600 MHz, CDCl_3_) *δ*: 3.89 (s, 3H, Ar-4’-OCH_3_), 3.95 (s, 3H, Ar-5’-OCH_3_), 5.54 (s, 2H, Ar-2-CH_2_-), 6.73 (d, *J* = 8.4 Hz, 1H, Ar-3-H), 6.96 (s, 1H, Ar-3’-H), 7.02 (s, 1H, Ar-6’-H), 7.03 (s, 1H, imidazole-4’’-H), 7.24 (s, 1H, imidazole-5’’-H), 7.35–7.36 (m, 3H, imidazole-2’’-Ph-H), 7.48 (d, *J* = 1.8 Hz, 1H, Ar-6-H), 7.49–7.50 (m, 2H, imidazole-2’’-Ph-H), 7.59 (dd, *J* = 1.8, 8.4 Hz, 1H, Ar-4-H); 555.14, 557.17, 558.99 ([M + H]^+^, 52, 100, 48).

**Compound 39a:** White solid, yield 79%, m.p. 164.2–165.1 °C; ^1^H-NMR (600 MHz, CDCl_3_) *δ*: 2.20 (s, 3H, imidazole-4’’-CH_3_), 2.26 (s, 3H, imidazole-2’’-CH_3_), 3.90 (s, 3H, Ar-4’-OCH_3_), 3.97 (s, 3H, Ar-5’-OCH_3_), 5.29 (s, 2H, Ar-2-CH_2_-), 6.55 (s, imidazole-5’’-H), 6.64 (d, *J* = 12.6 Hz, 1H, Ar-3-H), 7.01 (s, 1H, Ar-3’-H), 7.07 (s, 1H, Ar-6’-H), 7.48 (d, *J* = 3.0 Hz, 1H, Ar-6-H), 7.58 (dd, *J* = 3.0, 12.6 Hz, 1H, Ar-4-H); ESI-MS *m*/*z* (%) 507.22, 509.25, 511.10 ([M + H]^+^, 68, 100, 78).

**Compound 40a:** Yellow solid, yield 63%, m.p. 130.0–132.0 °C; ^1^H-NMR (600 MHz, CDCl_3_) *δ*: 3.78 (s, 3H, Ar-4’-OCH_3_), 3.95 (s, 3H, Ar-5’-OCH_3_), 5.68 (s, 2H, Ar-2-CH_2_-), 6.83 (s, 1H, Ar-3’-H), 6.89 (d, *J* = 12.6 Hz, 1H, Ar-3-H), 7.05 (s, 1H, Ar-6’-H), 7.23–7.24 (m, 2H, imidazole-Ar-H), 7.29–7.30 (m, 1H, imidazole-Ar-H), 7.49 (d, *J* = 3.0 Hz, 1H, Ar-6-H), 7.55 (dd, *J* = 3.0, 12.0 Hz, 1H, imidazole-Ar-H), 7.83 (d, *J* = 12.6 Hz, 1H, Ar-4-H ), 7.97 (s, 1H, imidazole-2’’-H); ESI-MS *m*/*z* (%) 529.16, 531.20, 533.04 ([M + H]^+^, 68, 100, 80).

#### 3.1.3. General Procedure for the Synthesis of Target Compounds **5b**–**40b**

BBr_3_ solution (BBr_3_/CH_2_Cl_2_, *v/v*, 1/9) 1.5 mL was dropwise added to a cooled (−78 °C) solution of 0.259 g (0.52 mmol) compound **5a** in 5 mL dried CH_2_Cl_2_. The mixture was allowed to warm to room temperature and stirred for 2 h, and poured into 30 mL ice-water. The precipitate was filtered, washed with a little distilled water and dried CH_2_Cl_2_, respectively, and dried in a vacuum drying oven to obtain 0.183 g yellow solid compound **5b** in 75% yield. The total yield of target compound **5b** was 18.8%.

Target compounds **6b**–**40b** were obtained from **6a** to **40a** in a similar manner as for the preparation of **5b** in 38–85% yield, the total yields of which were 7.7–23%。

**Compound 5b:** Yellow solid, final yield 18.8 %, m.p. 148.0–150.0 °C; ^1^H-NMR (600 MHz, DMSO-*d*_6_) *δ*: 1.73–1.82 (m, 6H, piperidine-3’’,4’’,5’’-H), 3.08 (t, *J* = 10.2 Hz 2H, piperidine-2’’-H), 3.43 (t, *J* = 12 Hz, 2H, piperidine-6’’-H), 4.37 (s, 2H, Ar-2-CH_2_-), 6.95 (s, 1H, Ar-6’-H), 7.08 (s, 1H, Ar-3’-H), 7.58 (s, 1H, Ar-6-H), 7.77 (d, *J* = 7.8 Hz, 1H, Ar-3-H), 7.98 (d, *J* = 8.4 Hz, 1H, Ar-4-H), 9.73 (brs, 1H, Ar-4’-OH), 10.41 (brs, 1H, Ar-5’-OH); ^13^C-NMR (150 MHz, DMSO-*d*_6_) *δ*: 21.6 × 2, 22.7 × 2, 53.1 × 2, 56.9, 110.7, 119.7, 120.9, 123.1, 129.0, 129.7, 133.8, 135.4, 135.6, 141.4, 145.3, 151.0, 194.9; ESI-MS *m*/*z* (%): 468.11, 470.13, 471.96 ([M + H]^+^, 78, 100, 98).

**Compound 6b:** Yellow solid, final yield 17.3%, m.p. 172.5–174.3 °C; ^1^H-NMR (600 MHz, DMSO-*d*_6_) *δ*: 1.48 (d, *J* = 6.0 Hz, 3H, piperidine-2’’-CH_3_), 1.69–1.92 (m, 6H, piperidine-3’’,4^’’^,5^’’^-H), 2.90–2.96 (m, 1H, piperidine-2’’-H), 3.12 (t, *J* = 4.8 Hz, 2H, piperidine-6’’-H), 4.83 (s, 1H, Ar-2-CH_2_-), 6.95 (s, 1H, Ar-6’-H), 7.08 (s, 1H, Ar-3’-H), 7.61 (d, *J* = 23.4 Hz, 1H, Ar-6-H), 7.79 (d, *J* = 8.4 Hz, 1H, Ar-3-H), 7.97 (d, *J* = 8.4 Hz, 1H, Ar-4-H), 9.72 (brs, 1H, Ar-4’-OH), 10.40 (brs, 1H, Ar-5’-OH); ^13^C-NMR (150 MHz, DMSO-*d*_6_) *δ*: 18.1, 22.4, 28.0, 30.9, 51.3, 57.6, 61.6, 110.8, 119.8, 121.0, 123.0, 128.9, 129.9, 133.8, 135.4, 135.9, 141.5, 145.2, 151.0, 194.8; ESI-MS *m*/*z* (%): 482.16, 484.19, 486.05 ([M + H]^+^, 82, 80, 100).

**Compound 7b:** Yellow solid, final yield 16.5%, m.p. 174.0–175.3 °C; ^1^H-NMR (600 MHz, DMSO-*d*_6_) *δ*: 0.88 (d, *J* = 6.6 Hz, 3H, piperidine-3’’-CH_3_), 1.06–1.13 (m, 1H, piperidine-3’’-H), 1.71–1.84 (m, 4H, piperidine-4’’,5’’-H), 2.66–2.71 (m, 2H, piperidine-2’’-H), 3.32–3.45 (m, 2H, piperidine-6’’-H), 4.38 (s, 2H, Ar-2-CH_2_), 6.96 (s, 1H, Ar-6’-H), 7.08 (s, 1H, Ar-3’-H), 7.58 (s, 1H, Ar-6-H), 7.80 (d, *J* = 8.4 Hz, 1H, Ar-3-H), 7.98 (d, *J* = 8.4 Hz, 1H, Ar-4-H), 9.71 (brs, 1H, Ar-4’-OH), 10.40 (brs, 1H, Ar-5’-OH); ^13^ C-NMR (150 MHz, DMSO-*d*_6_) *δ*: 19.0, 22.8, 29.1, 30.2, 52.7, 57.3, 58.4, 110.7, 119.8, 121.0, 123.2, 128.9, 129.5, 133.7, 135.4, 135.7, 141.5, 145.3, 151.0, 194.8; ESI-MS *m*/*z* (%): 482.15, 484.16, 485.98 ([M + H]^+^, 80, 95, 100).

**Compound 8b:** Yellow solid, final yield 15.9%, m.p. 103.3–105.0 °C; ^1^H-NMR (600 MHz, DMSO-*d*_6_) *δ*: 0.92 (d, *J* = 9.6 Hz, 3H, piperidine-4’’-CH_3_), 1.01–1.03 (m, 1H, piperidine-4’’-H), 1.38–1.47 (m, 2H, piperidine-3’’-H), 1.78–1.81 (m, 2H, piperidine-5’’-H), 3.09 (t, *J* = 18.6 Hz, 2H, piperidine-2’’-H), 3.44 (t, *J* = 16.2 Hz, 2H, piperidine-6’’-H), 4.37 (s, 2H, Ar-2-CH_2_-), 6.95 (s, 1H, Ar-6’-H), 7.08 (s, 1H, Ar-3’-H), 7.58 (d, *J* = 3.0 Hz, 1H, Ar-6-H), 7.79 (d, *J* = 12.0 Hz, 1H, Ar-3-H), 7.97 (dd, *J* = 12 Hz, 3.0 Hz, 1H, Ar-4-H), 9.75 (brs, 1H, Ar-4’-OH), 10.42 (brs, 1H, Ar-5’-OH); ^13^C-NMR (150 MHz, DMSO-*d*_6_) 21.5, 28.4, 31.2 × 2, 53.0 × 2, 57.2, 110.8, 113.8, 119.7, 120.9, 123.1, 129.8, 133.8, 135.6, 141.5, 145.3, 151.0, 156.3, 195.7; ESI-MS *m*/*z* (%): 481.99, 484.02, 485.92 ([M + H]^+^, 50, 100, 58).

**Compound 9b:** Yellow solid, final yield 15.3%, m.p. 173.0–175.3 °C; ^1^H-NMR (600 MHz, DMSO-*d*_6_) *δ*: 0.88 (d, *J* = 6.6 Hz, 6H, piperidine-3’’,5’’-CH_3_), 1.74 (t, *J* = 12.6 Hz, 2H, piperidine-4’’-H), 1.93–1.94 (m, 2H, piperidine-3’’,5’’-H), 2.59–2.65 (m, 2H, piperidine-2’’-H), 3.29–3.37 (m, 2H, piperidine-6’’-H), 4.37 (s, 2H, Ar-2-CH_2_-), 6.97 (s, 1H, Ar-3’-H), 7.08 (s, 1H, Ar-6’-H), 7.59 (s, 1H, Ar-6-H), 7.81 (d, *J* = 8.4 Hz, 1H, Ar-3-H), 7.98 (d, *J* = 8.4 Hz, 1H, Ar-4-H), 9.71 (brs, 1H, Ar-4’-OH), 10.41 (brs, 1H, Ar-5’-OH); ^13^C-NMR (150 MHz, DMSO-*d*_6_) 18.3 × 2, 28.3, 38.4 × 2, 56.7 × 2, 57.4, 110.3, 119.3, 120.5, 122.7, 128.3, 128.9, 133.2, 134.9, 135.4, 141.1, 144.7, 150.5, 194,3; ESI-MS *m*/*z* (%) 496.17, 498.42, 500.07 ([M + H]^+^, 82, 78, 100).

**Compound 10b:** Yellow solid, final yield 12.3%, m.p. 158.3–159.5 °C; ^1^H-NMR (600 MHz, DMSO-*d*_6_) *δ*: 1.79–2.12 (m, 5H, piperidine-3’’,4’’,5’’-H,), 3.14 (t, *J* = 12.6 Hz, 2H, piperdine-2’’-H), 3.50 (t, 2H, *J* = 12.0 Hz, 2H, piperidine-6’’-H), 4.38 (s, 2H, Ar-2-CH_2_-), 6.95 (s, 1H, Ar-3’-H), 7.08 (s, 1H, Ar-6’-H), 7.58 (s, 1H, Ar-6-H), 7.78 (d, *J* = 8.4 Hz, 1H, Ar-3-H), 7.98 (d, *J* = 7.8 Hz, 1H, Ar-4-H), 9.72 (s, 1H, Ar-4’-OH), 10.40 (s, 1H, Ar-5’-OH), 12.57 (s, 1H, piperidine-4’’-COOH); ^13^C-NMR (150 MHz, DMSO-*d*_6_) 25.7, 38.1 × 2, 52.1 × 2, 57.1, 110.7, 120.0, 120.9, 123.2, 129.0, 129.6, 133.8, 135.4, 135.6, 141.3, 145.3, 151.0, 175.0, 194.8; ESI-MS *m*/*z* (%) 512.08, 514.12, 515.93 ([M + H]^+^, 65, 100, 75).

**Compound 11b:** Yellow solid, final yield 15.7%, m.p. 180.0–181.3 °C; FT-IR (ATR) υ (cm^−1^): 3191, 2979, 2878, 1716, 1652, 1586, 1401, 1287, 1188, 1012, 796, 639; ^1^H-NMR (600 MHz, DMSO-*d*_6_) *δ*: 1.20 (t, *J* = 10.8 Hz, 3H, piperidine-3’’-COOCH_2_-CH_3_), 1.50–2.04 (m, 4H, piperidine-5’’,6’’-H), 3.07–3.60 (m, 5H, piperidine-2’’,3’’,4’’-H), 4.10 (q, 2H, piperidine-3’’-COOCH_2_-), 4.45 (s, 2H, Ar-2-CH_2_-), 6.96 (s, 1H, Ar-3’-H), 7.08 (s, 1H, Ar-6’-H), 7.59 (d, *J* = 3.0 Hz, 1H, Ar-6-H), 7.81 (d, *J* = 12.0 Hz, 1H, Ar-3-H), 7.98 (d, *J* = 12.0 Hz, 1H, Ar-4-H), 9.74 (brs, 1H, Ar-4’-OH), 10.43 (brs, 1H, Ar-5’-OH); ^13^C-NMR (150 MHz, DMSO-*d*_6_) 14.4, 22.2, 25.1, 52.6, 55.4, 57.7, 61.2, 61.5, 110.8, 113.8, 119.7, 120.9, 123.3, 129.0, 133.8, 135.7, 141.5, 145.3, 151.0, 156.3, 171.4, 194.8; ESI-MS *m*/*z* (%) 539.96, 541.95, 543.78 ([M + H]^+^, 52, 100, 70); HR-MS (ESI) calcd for C_22_H_23_Br_2_NO_5_ [M − H]^−^: 539.9840; found: 539.9835.

**Compound 12b:** Yellow solid, final yield 19.0%, m.p. 184.0–186.0 °C; FT-IR (ATR) υ (cm^−1^): 3191, 2960, 2764, 1649, 1586, 1411, 1286, 1191, 1152, 1010, 796, 638; ^1^H-NMR (600 MHz, DMSO-*d*_6_) *δ*: 1.75–1.99 (m, 4H, piperidine-3’’,5’’-H), 3.12-3.45 (m, 4H, piperidine-2’’,6’’-H), 3.94 (s, 1H, piperidine-4’’-OH), 3.67–3.72 (m, 1H, piperidine-4’’-H), 4.39 (s, 2H, Ar-2-CH_2_-), 6.96 (s, 1H, Ar-3’-H), 7.09 (s, 1H, Ar-6’-H), 7.58 (d, *J* = 3.0 Hz, 1H, Ar-6-H), 7.84 (d, *J* = 12.0 Hz, 1H, Ar-3-H), 7.98 (d, *J* = 12.6 Hz, 1H, Ar-4-H), 9.78 (s, 1H, Ar-4’-OH), 10.37 (s, 1H, Ar-5’-OH); ^13^C-NMR (150 MHz, DMSO-*d*_6_) 29.8 × 2, 48.1 × 2, 59.9, 64.2, 110.6, 119.6, 120.9, 123.2, 129.1, 129.7, 133.9, 135.5, 135.8, 141.4, 145.3, 150.9, 195.0; ESI-MS *m*/*z* (%) 483.74, 485.71, 487.62 ([M + H]^+^, 48, 100, 58); HR-MS (ESI) calcd for C_19_H_19_Br_2_NO_4_ [M + H]^+^: 485.9730; found: 485.9692.

**Compound 13b:** Yellow solid, final yield 15.3%, m.p. 98.0–99.5 °C; FT-IR (ATR) υ (cm^−1^): 3191, 2975, 2941, 1655, 1586, 1409, 1279, 1191, 1118, 1009, 805, 637; ^1^H-NMR (600 MHz, DMSO-*d*_6_) *δ*: 1.20 (d, *J* = 9.0 Hz, 6H, piperidine-2’’,6’’-CH_3_), 3.40–3.50 (m, 6H, piperidine-3’’,4’’,5’’-H), 4.63–4.67 (m, 2H, piperidine-2’’,6’’-H), 5.76 (s, 2H, Ar-2-CH_2_-), 6.98 (s, 1H, Ar-3’-H), 7.10 (s, 1H, Ar-6’-H), 7.58 (d, *J* = 2.4 Hz, 1H, Ar-3-H), 7.79 (d, *J* = 5.4 Hz, 1H, Ar-6-H), 7.98 (d, *J* = 3.0 Hz, 1H, Ar-4-H), 9.75 (brs, 1H, Ar-4’-OH), 10.48 (brs, 1H, Ar-5’-OH); ^13^C-NMR (150 MHz, DMSO-*d*_6_) 18.4, 19.5 × 2, 30.1 × 2, 52.8 × 2, 55.4, 110.8, 119.8, 121.1, 122.2, 128.9, 132.7, 133.7, 134.3, 135.3, 139.4, 145.1, 151.0, 195.1; ESI-MS *m*/*z* (%) 494.20, 496.18, 498.15 ([M − H]^−^, 50, 100, 50); HR-MS (ESI) calcd for C_21_H_23_Br_2_NO_3_ [M − H]^−^: 495.9940, found: 495.9961.

**Compound 14b:** Yellow solid, final yield 5.3%, m.p. 176.0–177.3 °C; FT-IR (ATR) υ (cm^−1^): 2951, 2921, 2850, 1654, 1606, 1442, 1394, 1283, 1048, 999, 864, 686; ^1^H-NMR (600 MHz, DMSO-*d*_6_) *δ*: 0.87 (s, 12H, piperidine-2’’,6’’-CH_3_), 1.23–1.29 (m, 6H, piperidine-3’’,4’’,5’’-H), 3.77 (s, 2H, Ar-2-CH_2_-), 6.67 (s, 1H, Ar-3’-H), 6.92 (s, 1H, Ar-6’-H), 7.34 (d, *J* = 3.0 Hz, 1H, Ar-6-H), 7.69 (d, *J* = 12.6 Hz, 1H, Ar-3-H), 7.95 (d, *J* = 12.6 Hz, 1H, Ar-4-H), 9.74 (brs, 1H, Ar-4’-OH), 10.38 (brs, 1H, Ar-5’-OH); ^13^C-NMR (150 MHz, DMSO-*d*_6_) 17.8, 41.2 × 4, 45.5 × 2, 54.8 × 2, 54.9, 109.7, 110.3, 113.5, 118.5, 125.0, 129.6, 131.3, 131.4, 133.7, 139.2, 144.9, 155.8, 196.5; ESI-MS *m*/*z* (%) 523.98, 525.96, 527.82 ([M + H]^+^, 48, 100, 58); HR-MS (ESI) calcd for C_25_H_31_Br_2_NO_4_-H: 524.026, found: 524.0268.

**Compound 15b:** Yellow solid, final yield 12.7%, m.p. 122.5–124.0 °C; ^1^H-NMR (600 MHz, DMSO-*d*_6_) *δ*: 1.52–1.90 (m, 6H, piperidine-3’’, 4’’, 5’’-H), 3.03–3.13 (m, 2H, piperidine-6’’-H), 3.34 (s, 1H, piperidine-2’’-H), 3.74–3.92 (m, 2H, piperidine-2’’-CH_2_-), 4.37 (s, 2H, Ar-2-CH_2_-), 6.96 (s, 1H, Ar-3’-H), 7.08 (s, 1H, Ar-6’-H), 7.56 (s, 1H, Ar-6-H), 7.78 (d, *J* = 12.6 Hz, 1H, Ar-3-H), 7.99 (d, *J* = 12 Hz, 1H, Ar-4-H), 9.78 (brs, 1H, Ar-4’-OH), 10.46 (brs, 1H, Ar-5’-OH); ^13^C-NMR (150 MHz, DMSO-*d*_6_) 20.8, 21.7, 25.4, 50.7, 53.3, 55.4, 59.6, 111.0, 119.9, 121.0, 123.1, 128.8, 129.8, 133.8, 135.4, 135.9, 141.7, 145.2, 151.1, 195.2; ESI-MS *m*/*z* (%) 497.74, 499.67, 501.64 ([M + H]^+^, 75, 100, 70).

**Compound 16b:** Yellow solid, final yield 16.2%, m.p. 50.0–51.0 °C; FT-IR (ATR) υ (cm^−1^): 3360, 2920, 2851, 1631, 1588, 1500, 1362, 1289, 1237, 1153, 1012, 878, 798; ^1^H-NMR (600 MHz, DMSO-*d*_6_) *δ*: 1.04–1.46 (m, 7H, piperidine-3’’,4’’,5’’-H, piperidine-4’’-CH_2_), 3.03–3.45 (m, 6H, piperidine-2’’,6’’-H, HO-CH_2_-), 4.37 (s, 2H, Ar-2-CH_2_-), 6.96 (s, 1H, Ar-3’-H), 7.08 (s, 1H, Ar-6’-H), 7.57 (d, *J* = 3.0 Hz, 1H, Ar-6-H), 7.81 (d, *J* = 12.6 Hz, 1H, Ar-3-H), 7.98 (dd, *J* = 3.0, 12.6 Hz, 1H, Ar-4-H), 9.74 (brs, 1H, Ar-4’-OH), 10.41 (brs, 1H, Ar-5’-OH); ^13^C-NMR (150 MHz, DMSO-*d*_6_) 29.3, 30.2 × 2, 38.8 × 2, 53.1, 57.1, 58.4, 110.7, 119.7, 120.9, 123.1, 129.0, 129.7, 133.7, 135.4, 135.6, 141.5, 145.3, 150.9, 194.8; ESI-MS *m*/*z* (%) 511.96, 513.95, 515.79 ([M + H]^+^, 55, 100, 70).

**Compound 17b:** White solid, fianl yield 13.5%, m.p. 113.0–115.0 °C; ^1^H-NMR (600 MHz, DMSO-*d*_6_) *δ*: 1.42–1.85 (m, 5H, piperidine-3’’,4’’,5’’-H), 3.04–3.13 (m, 2H, piperidine-2’’-H), 3.28 (d, *J* = 8.4 Hz, 2H, HO-CH_2_-), 3.42–3.57 (m, 2H, piperidine-6’’-H), 4.39 (s, 2H, Ar-2-CH_2_-), 5.76 (s, 1H, -OH), 6.96 (d, *J* = 5.4 Hz, 1H, Ar-3’-H), 7.08 (s, 1H, Ar-6’-H), 7.57 (s, 1H, Ar-6-H), 7.78 (dd, *J* = 3.6, 12.6 Hz, 1H, Ar-3-H), 7.98 (d, *J* = 12.6 Hz, 1H, Ar-4-H), 9.75 (brs, 1H, Ar-4’-OH), 10.42 (brs, 1H, Ar-5’-OH); ^13^C-NMR (150 MHz, DMSO-*d*_6_) 26.2 × 2, 36.1, 52.8×2, 57.2, 65.2, 110.8, 119.7, 120.9, 123.1, 129.0, 129.7, 133.8, 135.4, 135.5, 141.5, 145.2, 151.0, 194.8; ESI-MS *m*/*z* (%) 497.91, 499.94, 501.79 ([M + H]^+^, 60, 100, 75).

**Compound 18b:** White solid, final yield 10.0%, m.p. 183.0–185.0 °C; ^1^H-NMR (400 MHz, DMSO-*d*_6_) *δ*: 3.34 (s, 8H, piperazine-2’’,3’’,5’’,6’’-H), 4.29 (s, 4H, Ar-2-CH_2_-), 6.96 (s, 2H, Ar-3’-H), 7.09 (s, 2H, Ar-6’-H), 7.57 (s, 2H, Ar-6-H), 7.76 (d, *J* = 9.6 Hz, 2H, Ar-3-H), 7.94 (d, *J* = 9.0 Hz, 2H, Ar-4-H), 9.90 (brs, 4H, Ar-4’-OH, Ar-5’-OH); ^13^ C-NMR (150 MHz, DMSO-*d*_6_) 49.6 × 4, 57.1 × 2, 110.7 × 2, 119.7 × 2, 121.0 × 2, 122.9 × 2, 126.3 × 2, 128.7 × 2, 133.5 × 2, 135.1 × 2, 141.5 × 2, 145.2 × 2, 150.9 × 2, 156.2 × 2, 194.7 × 2; ESI-MS *m*/*z* (%) 850.69, 852.83, 854.92 ([M + H]^+^, 52, 100, 48).

**Compound 19b:** Yellow solid, final yield 18.4%, m.p. 195.8–197.5 °C; ^1^H-NMR (600 MHz, DMSO-*d*_6_) *δ*: 1.20–1.28 (m, 5H, piperazine-CH_2_CH_3_), 3.16 (m, 4H, piperazine- 3’’,5’’-H), 3.53 (m, 4H, piperazine-2’’,6’’-H), 3.88 (s, 2H, Ar-2-CH_2_-), 6.95 (s, 1H, Ar-3’-H), 7.06 (s, 1H, Ar-6’-H), 7.09 (s, 1H, Ar-3-H), 7.14 (s, 1H, Ar-4-H), 7.23 (s, 1H, Ar-6-H), 9.02 (brs, 1H, Ar-4’-OH), 9.67 (brs, 1H, Ar-5’-OH); ^13^ C-NMR (150 MHz, DMSO-*d*_6_) 9.3, 25.7, 31.7 × 2, 50.5 × 2, 52.3, 110.7, 116.9, 119.8, 121.2, 128.5, 132.9, 134.8, 141.6, 145.1, 148.5, 150.8, 152.5, 194.5; ESI-MS *m*/*z* (%) 497.11, 499.11, 500.96 ([M + H]^+^, 48, 100, 55).

**Compound 20b:** Yellow solid, fianl yield 18.1%, m.p. 219.5–220.8 °C; ^1^H-NMR (600 MHz, DMSO-*d*_6_) *δ*: 3.15 (s, 2H, piperazine-2’’-H), 3.35 (s, 2H, piperazine-6’’-H), 3.54 (s, 2H, piperazine-3’’-H), 3.84 (s, 2H, piperazine-5’’-H), 4.51 (s, 2H, Ar-2-CH_2_-), 6.87-7.01 (m, 5H, piperazine-C_6_H_5_), 7.27 (s, 2H, Ar-3’,6’-H), 7.60 (s, 1H, Ar-3-H), 7.83 (s, 1H, Ar-4-H), 8.00 (s, 1H, Ar-6-H), 9.78 (brs, 1H, Ar-4’-OH), 10.38 (brs, 1H, Ar-5’-H); ^13^C-NMR (150 MHz, DMSO-*d*_6_) 45.5 × 2, 51.5 × 2, 56.9, 110.7, 111.7, 118.4 × 2, 119.7, 120.9, 123.3, 129.0, 129.6, 132.2 × 2, 133.9, 135.5, 135.7, 141.4, 145.3, 149.1, 151.0, 195.0; ESI-MS *m*/*z* (%) 545.11, 547.15, 548.96 ([M + H]^+^, 60, 100, 80).

**Compound 21b:** White solid, fianl yield 15.9%, m.p. 156.0–157.8 °C; FT-IR (ATR) υ (cm^−1^): 3189, 2636, 2251, 2261, 1654, 1588, 1500, 1411, 1191, 1014, 752, 637; ^1^H-NMR (600 MHz, DMSO-*d*_6_) *δ*: 3.02–3.53 (m, 8H, piperazine-2’’,3’’,5’’,6’’-H), 3.80 (s, 3H, Ar-OCH_3_), 4.50 (s, 2H, Ar-2-CH_2_-), 6.92–7.04 (m, 5H, piperazine-C_6_H_4_-, Ar-3’-H), 7.09 (s, 1H, Ar-6’-H), 7.60 (s, 1H, Ar-6-H), 7.82 (d, *J* = 12 Hz, 1H, Ar-6-H), 8.01 (d, *J* = 12.6 Hz, 1H, Ar-4-H), 9.29 (brs, 2H, Ar-4’-OH, Ar-5’-OH); ^13^C-NMR (150 MHz, DMSO-*d*_6_) 47.3, 52.2 × 2, 52.3 × 2, 56.0, 110.7, 112.5, 115.5, 118.9, 119.7, 120.9, 121.4, 123.3, 124.1, 129.0, 129.4, 133.9, 135.5, 135.9, 141.1, 145.3, 150.9, 152.3, 195.0; ESI-MS *m*/*z* (%) 560.71, 562.71, 564.86 ([M + H]^+^, 45, 100, 55); HR-MS (ESI) calcd for C_25_H_24_Br_2_N_2_O_4_ [M − H]^−^: 575.0000, found: 575.0241.

**Compound 22b:** Yellow solid, fianl yield 14.2%, m.p. 208.0–209.0 °C; FT-IR (ATR) υ (cm^−1^): 3193, 2770, 2708, 2262, 1649, 1587, 1410, 1280, 1191, 1151, 1010, 796, 638; ^1^H-NMR (600 MHz, DMSO-*d*_6_) *δ*: 2.05 (s, 3H, piperazine-CO-CH_3_), 3.08–3.50 (m, 8H, piperazine-2’’,3’’, 5’’,6’’-H,), 4.48 (s, 2H, Ar-2-CH_2_-), 6.99 (s, 1H, Ar-3’-H), 7.10 (s, 1H, Ar-6’-H), 7.58 (d, *J* = 3.6 Hz, 1H, Ar-6-H), 7.86 (d, *J* = 12.6 Hz, 1H, Ar-3-H), 8.00 (dd, *J* = 3.0, 12.6 Hz, 1H, Ar-4-H), 9.08 (brs, 1H, Ar-4’-OH), 9.62 (brs, 1H, Ar-5’-OH); ^13^C-NMR (150 MHz, DMSO-*d*_6_) 21.4, 42.9 × 2, 52.0 × 2, 56.9, 110.8, 119.7, 120.9, 123.3, 129.0, 129.3, 133.9, 135.5, 135.7, 141.4, 145.3, 151.0, 169.1, 194.9; ESI-MS *m*/*z* (%) 510.72, 512.69, 514.70 ([M + H]^+^, 48, 100, 52); HR-MS (ESI) calcd for C_20_H_20_Br_2_N_2_O_4_ [M − H]^−^: 510.969, found: 510.9676.

**Compound 23b:** Yellow solid, final yield 8.3%, m.p. 64.5–66.3 °C; ^1^H-NMR (600 MHz, DMSO-*d*_6_) *δ*: 3.35–3.88 (m, 8H, piperazine-2’’,3’’,5’’,6’’-H), 4.49 (s, 2H, Ar-2-CH_2_-), 6.96 (s, 1H, Ar-3’-H), 7.07–8.20 (m, 8H, piperazine-C_6_H_4_-, Ar-6’,3,4,6-H), 9.75 (brs, 1H, Ar-4’-OH), 10.43 (brs, 1H, Ar-5’-OH); ^13^C-NMR (150 MHz, DMSO-*d*_6_) 19.0 × 2, 51.5 × 2, 56.5, 110.8 × 2, 112.9 × 2, 113.9, 119.8, 120.9, 123.4, 126.2, 129.0, 129.4, 132.2, 133.9, 135.6, 135.7, 141.5, 145.2, 150.9, 195.0; ESI-MS *m*/*z* (%) 590.19, 591.91, 593.79 ([M + H]^+^, 70, 100, 50).

**Compound 24b:** Yellow solid, final yield 16.1%, m.p. 217.0–218.5 °C; FT-IR (ATR) υ (cm^−1^): 3363, 3010, 2699, 1633, 1585, 1500, 1411, 1365, 1298, 1119, 1031, 798, 639;^1^H-NMR (600 MHz, DMSO-*d*_6_) *δ*: 2.86 (s, 3H, piperazine-4’’-CH_3_), 3.64–3.77 (m, 8H, piperazine-2’’,3’’, 5’’,6’’-H), 4.60 (s, 2H, Ar-2-CH_2_-), 6.97 (s, 1H, Ar-3’-H), 7.10 (s, 1H, Ar-6’-H), 7.55 (s, 1H, Ar-6-H), 7.91 (d, *J* = 9.0 Hz,1H, Ar-3-H), 7.98 (d, *J* = 10.2 Hz, 1H, Ar-4-H), 10.06 (brs, 2H, Ar-4’-OH, Ar-5’-OH); ^13^C-NMR (150 MHz, DMSO-*d*_6_) 19.0, 42.5, 49.1 × 2, 56.5 × 2, 110.6, 119.8, 121.1, 122.3, 128.5, 132.5, 132.8, 134.5, 141.7, 145.2, 150.8, 158.3, 194.4; ESI-MS *m*/*z* (%) 482.75, 484.62, 486.57 ([M + H]^+^, 50, 100, 40); HR-MS (ESI) calcd for C_19_H_20_Br_2_N_2_O_3_ [M + H]^+^: 484.9894, found: 484.9927.

**Compound 25b:** Yellow solid, fianl yield 14.4%, m.p. 208.0–210.0 °C; FT-IR (ATR) υ (cm^−1^): 3479, 3194, 3006, 2622, 2519, 2260, 1657, 1604, 1406, 1284, 1190, 1013, 798, 639; ^1^H-NMR (600 MHz, DMSO-*d*_6_) *δ*: 1.27 (d, *J* = 9.6 Hz, 6H, piperazine-4’’-CH_3_), 3.18–3.58 (m, 9H, piperazine-2’’,3’’,5’’,6’’-H, piperazine-CHMe_2_), 4.17 (s, 2H, Ar-2-CH_2_-), 6.97 (s, 1H, Ar-3’-H), 7.10 (s, 1H, Ar-6’-H), 7.56 (s, 1H, Ar-6-H), 7.75 (d, *J* = 12.0 Hz, 1H, Ar-3-H), 7.91 (d, *J* = 10.2 Hz, 1H, Ar-4-H), 10.00 (brs, 2H, Ar-4’-OH, Ar-5’-OH); ^13^C-NMR (150 MHz, DMSO-*d*_6_) 16.7×2, 45.9, 49.2×2, 56.7, 57.8 *×* 2, 110.7, 120.0, 121.2, 122.7, 126.0, 128.4, 130.7, 132.9, 134.7, 141.6, 145.1, 150.9, 194.5; ESI-MS *m*/*z* (%) 510.70, 512.77, 514.75 ([M + H]^+^, 55, 100, 57); HR-MS (ESI) calcd for C_21_H_24_Br_2_N_2_O_3_ [M + H]^+^: 513.0210, found: 513.0218.

**Compound 26b:** White solid, final yield 13.9%, m.p. 160.0–162.0 °C; FT-IR (ATR) υ (cm^−1^): 3194, 2798, 2524, 2361, 1652, 1588, 1412, 1292, 1192, 1015, 883, 639;^1^H-NMR (600 MHz, DMSO-*d*_6_) *δ*: 3.12–3.44 (m, 9H, piperazine-2’’,3’’,5’’,6’’-H, Ph_2_CH-), 4.39 (s, 2H, Ar-2-CH_2_-), 6.95 (s, 1H, Ar-3’-H), 7.09 (s, 1H, Ar-6’-H), 7.33–7.74 (m, 12H, Ar-H, Ar-4’’’-H), 7.92 (d, *J* = 10.2 Hz, 1H, Ar-4-H), 9.00 (brs, 2H, Ar-4’-OH, Ar-5’-OH); ^13^ C-NMR (150 MHz, DMSO-*d*_6_) 40.5 × 2, 48.9 × 2, 56.5, 73.6, 110.6, 112.8, 119.6, 120.9 × 2, 126.7, 127.2, 128.5 *×* 4, 128.9, 129.6 *×* 4, 130.0, 133.2, 133.7, 135.2×2, 141.3, 145.2, 150.8, 194.8; ESI-MS *m*/*z* (%) 634.80, 636.74, 638.50 ([M + H]^+^, 55, 100, 45); HR-MS (ESI) calcd for C_31_H_28_Br_2_N_2_O_3_ [M−H]^−^: 635.0370, found: 635.0389.

**Compound 27b:** Yellow solid, final yield 15.1%, m.p. 56.0–58.0 °C; ^1^H-NMR (600 MHz, DMSO-*d*_6_) *δ*: 2.22–2.31 (m, 10H, piperazine-2’’,3’’,5’’,6’’-H, Ar-2-CH_2_-), 3.31 (t, *J* = 10.8 Hz, 2H, piperazine-4’’-CH_2_-), 3.45 (t, *J* = 10.2 Hz, 2H, HO-CH_2_-), 6.92 (s, 1H, Ar-3’-H), 7.04 (s, 1H, Ar-6’-H), 7.37 (d, *J* = 12.0 Hz, 1H, Ar-3-H), 7.41 (d, *J* = 1.8 Hz, 1H, Ar-6-H), 7.66 (d, *J* = 12.6 Hz, 1H, Ar-4-H), 9.83 (brs, 2H, Ar-4’-OH, Ar -5’-OH); ^13^C-NMR (150 MHz, DMSO-*d*_6_) 52.9 *×* 2, 53.2 *×* 2, 58.8, 59.5, 60.6, 110.6, 111.6, 113.9, 120.3, 127.9, 131.7, 132.2, 133.2, 137.7, 142.5, 150.7, 155.9, 194.5; ESI-MS *m*/*z* (%) 512.92, 514.96, 516.81 ([M + H]^+^, 60, 100, 70).

**Compound 28b:** Yellow solid, final yield 18.6%, m.p. 114.0–115.0 °C; ^1^H-NMR (600 MHz, DMSO-*d*_6_) *δ*: 3.28–3.57 (m, 6H, piperazine-H), 4.49 (s, 2H, Ar-2-CH_2_-), 4.72 (d, *J* = 14.4 Hz, 2H, piperazine-H), 6.80 (t, *J* = 7.2 Hz, 1H, pyrimidine-5’’’-H), 7.00 (s, 1H, Ar-3’-H), 7.10 (s, 1H, Ar-6’-H), 7.58 (d, *J* = 2.4 Hz, 1H, Ar-6-H), 7.91 (d, 1H, *J* = 12.6 Hz, Ar-3-H), 8.01 (dd, *J* = 12.6 Hz, 1H, Ar-4-H), 8.47 (d, *J* = 7.2 Hz, 2H, pyrimidine-4’’’,6’’’-H), 9.70 (brs, 2H, Ar-4’-OH, Ar-5’-OH); ^13^C-NMR (150 MHz, DMSO-*d*_6_) 40.8 *×* 2, 51.5 *×* 2, 57.0, 110.7, 111.8, 119.7, 120.9, 123.3, 129.0, 129.4, 133.8, 135.5, 135.7, 141.5, 145.3, 150.9, 158.6 *×* 2, 160.8, 194.9; ESI-MS *m*/*z* (%) 546.72, 548.73, 550.59 ([M + H]^+^, 48, 100, 60).

**Compound 29b:** White solid, final yield 21.1%, m.p. 193.0–195.0 °C; ^1^H-NMR (600 MHz, DMSO-*d*_6_) *δ*: 3.08–3.77 (m, 8H, piperazine-2’’,3’’,5’’,6’’-H), 4.50 (s, 2H, Ar-2-CH_2_-), 6.97 (s, 1H, Ar-3’-H), 7.03 (dd, *J* = 6.6, 13.8 Hz, 2H, piperazine-Ar-2’’’,6’’’-H), 7.09 (s, 1H, Ar-6’-H), 7.12 (d, *J* = 13.2 Hz, 2H, piperazine-Ar-3’’’,5’’’-H), 7.60 (d, 1H, *J* = 2.4 Hz, Ar-6-H), 7.83 (d, *J* = 12.6 Hz, 1H, Ar-3-H), 8.01 (dd, *J* = 3.0, 12.6 Hz, 1H, Ar-4-H), 9.39 (brs, 2H, Ar-4’-OH, Ar-5’-OH); ^13^C-NMR (150 MHz, DMSO-*d*_6_) 46.5 × 2, 51.7 × 2, 56.7, 110.7, 116.1 × 2, 118.4, 119.7, 120.9, 123.3, 129.0, 129.4, 133.9, 135.5 × 2, 141.5, 145.3, 146.7, 151.0, 156.3, 157.9, 194.9; ESI-MS *m*/*z* (%) 562.96, 564.95, 566.77 ([M + H]^+^, 48, 100, 60).

**Compound 30b:** Yellow solid, final yield 19.4%, m.p. 190.0–191.5 °C; ^1^H-NMR (600 MHz, DMSO-*d*_6_) *δ*: 3.14–3.57 (m, 8H, piperazine-2’’,3’’,5’’ 6’’,-H), 4.52 (s, 2H, Ar-2-CH_2_-), 6.97 (s, 1H, Ar-3’-H), 7.05–7.22 (m, 5H, Ar-6’-H, piperazine-H), 7.60 (s, 1H, Ar-6-H), 7.80 (dd, *J* = 6.6, 12.6 Hz, 1H, Ar-3-H), 8.01 (d, *J* = 12.6 Hz, 1H, Ar-4-H), 9.35 (brs, 2H, Ar-4’-OH, Ar-5’-OH); ^13^C-NMR (150 MHz, DMSO-*d*_6_) 47.4 × 2, 52.0 × 2, 57.0, 110.7, 116.7, 119.7, 120.2, 120.9, 123.3, 124.0, 125.5, 129.1, 129.4, 134.0, 135.8, 138.7, 141.4, 145.3, 151.0, 154.5, 156.2, 195.0; ESI-MS *m*/*z* (%) 562.97, 564.95, 566.79 ([M + H]^+^, 60, 100, 75).

**Compound 31b:** White solid, final yield 17.5%, m.p. 185.0–186.0 °C; ^1^H-NMR (600 MHz, DMSO-*d*_6_) *δ*: 3.04–3.56 (m, 8H, piperazine-2’’,3’’,5’’,6’’-H), 4.49 (s, 2H, Ar-2-CH_2_-), 6.72 (d, 2H, *J* = 12.0 Hz,2H, piperazine-Ar-2’’’,6’’’-H), 6.88 (d, *J* = 12.0 Hz, 2H, piperazine-Ar-3’’’,5’’’-H), 6.97 (s, 1H, Ar-3’-H), 7.09 (s, 1H, Ar-6’-H), 7.59 (s, 1H, Ar-6-H), 7.80 (s, *J* = 12.0 Hz, 1H, Ar-3-H), 7.99 (d, *J* = 12.0 Hz, 1H, Ar-4-H), 9.32 (brs, 3H, Ar-4’-OH, Ar-5’-OH, Ar-4’’’-OH); ^13^C-NMR (150 MHz, DMSO-*d*_6_) 40.5 × 2, 48.9 × 2, 56.5, 110.7, 116.1 × 2, 119.9, 120.9, 124.4, 125.4, 126.1, 136.7, 137.1, 138.0, 141.5, 145.3, 150.2 × 2, 150.9, 152.3, 153.4, 184.5; ESI-MS *m*/*z* (%) 560.98, 562.97, 564.81 ([M + H]^+^, 45, 100, 60).

**Compound 32b:** Yellow solid, final yield 20.5%, m.p. 205.8–207.3 °C; ^1^H-NMR (600 MHz, DMSO-*d*_6_) *δ*: 5.58 (s, 2H, Ar-2-CH_2_-), 6.93 (s, 1H, Ar-3’-H), 7.06 (s, 1H, Ar-6’-H), 7.45 (d, *J* = 7.8 Hz, 1H, Ar-3-H), 7.52 (s, 1H, Ar-6-H), 7.71 (t, *J* = 11.4 Hz, 2H, imidazole-4’’,5’’-H), 7.88 (d, *J* = 7.8 Hz, 1H, Ar-4-H), 9.14 (t, *J* = 11.4 Hz, 1H, imidazole-2’’-H), 9.70 (brs, 1H, Ar-4’-OH), 10.39(brs, 1H, Ar-5’-OH); ^13^C-NMR (150 MHz, DMSO-*d*_6_) 49.7, 110.5, 119.4, 119.9, 120.6, 120.8, 122.3, 122.8, 129.0, 133.2, 133.5, 135.6, 136.5, 140.0, 145.2, 150.8, 194.7; ESI-MS *m*/*z* (%) 451.00, 453.06, 454.88 ([M + H]^+^, 70, 100, 80).

**Compound 33b:** White solid, final yield 21.1%, m.p. 217.0–218.5 °C; ^1^H-NMR (600 MHz, DMSO-*d*_6_) *δ*: 2.53 (s, 3H, imidazole-2’’-CH_3_), 5.53 (s, 2H, Ar-2-CH_2_-), 6.95 (s, 1H, Ar-3’-H), 7.08 (s, 1H, Ar-6’-H), 7.19 (d, *J* = 12.6 Hz, 1H, Ar-3-H), 7.50 (d, *J* = 3.0 Hz, 1H, Ar-6-H), 7.53 (d, *J* = 3.0 Hz, 1H, imidazole-5’’-H), 7.62 (d, *J* = 3.0 Hz, 1H, imidazole-4’’-H), 7.85 (dd, *J* = 3.0, 12.6 Hz, 1H, Ar-4-H), 9.75 (brs, 1H, Ar-4’-OH), 10.39 (brs, 1H, Ar-5’-OH); ^13^C-NMR (150 MHz, DMSO-*d*_6_) 11.1, 48.4, 110.5, 118.8, 119.5, 120.9, 121.9, 123.0, 128.8, 131.9, 132.0, 133.5, 133.7, 135.5, 139.4, 145.3, 150.9, 194.6; ESI-MS *m*/*z* (%) 464.69, 466.67, 468.57 ([M + H]^+^, 50, 100, 60).

**Compound 34b:** White solid, final yield 17.3%, m.p. 170.0–172.0 °C; ^1^H-NMR (600 MHz, DMSO-*d*_6_) *δ*: 2.20 (s, 3H, imidazole-4’’-CH_3_), 5.45 (s, 2H, Ar-2-CH_2_-), 6.90 (s, 1H, imidazole-5’’-H), 7.05 (s, 1H, Ar-3’-H), 7.27 (s, 1H, Ar-6’-H), 7.37 (d, *J* = 12.0 Hz, 1H, Ar-3-H), 7.50 (d, *J* = 3.0 Hz, 1H, Ar-6-H), 7.87 (dd, *J* = 3.0, 12.0 Hz, 1H, Ar-4-H), 8.76 (s, 1H, imidazole-2’’-H), 9.72 (brs, 1H, Ar-4’-OH), 10.42 br (s, 1H, Ar-5’-OH); ^13^C-NMR (150 MHz, DMSO-*d*_6_) 10.6, 49.3, 110.5, 118.9, 119.5, 120.9, 122.1, 128.9, 131.3, 133.0, 133.2, 134.2, 135.4, 135.9, 140.0, 145.2, 150.9, 194.7; ESI-MS *m*/*z* (%) 464.86, 466.88, 498.75 ([M + H]^+^, 54, 100, 65).

**Compound 35b:** White solid, final yield 20.5%, m.p. 235.2–236.9 °C; FT-IR (ATR) υ (cm^−1^): 3184, 3159, 2789, 2673, 2260, 1667, 1598, 1500, 1383, 1194, 1012, 882, 801, 640; ^1^H-NMR (600 MHz, DMSO-*d*_6_) *δ*: 1.20 (t, *J* = 11.4 Hz, 3H, imidazole-2’’-CH_3_), 2.90 (q, *J* = 11.4 Hz, 2H, imidazole-2’’-CH_2_-), 5.56 (s, 2H, Ar-2-CH_2_-), 6.94 (s, 1H, Ar-3’-H), 7.07 (s, 1H, Ar-6’-H), 7.16 (d, *J* = 6.0 Hz, 1H, Ar-6-H), 7.52 (d, *J* = 3.0 Hz, 1H, imidazole-5’’-H), 7.54 (d, *J* = 3.6 Hz, 1H, imidazole-4’’-H), 7.66 (d, *J* = 3.0 Hz, 1H, Ar-3-H), 7.84 (dd, *J* = 3.0, 12.6 Hz, 1H, Ar-4-H), 9.72 (brs, 1H, Ar-4’-OH), 10.35 (brs, 1H, Ar-5’-OH); ^13^C-NMR (150 MHz, DMSO-*d*_6_) 11.2, 18.5, 48.4, 110.5, 119.0, 119.5, 120.9, 121.9, 123.1, 128.8, 131.7, 133.6, 133.9, 135.5, 139.3, 145.3, 149.2, 150.9, 194.5; ESI-MS *m*/*z* (%) 478.78, 480.63, 482.73 ([M + H]^+^, 48, 100, 45); HR-MS (ESI) calcd for C_19_H_16_Br_2_N_2_O_3_ [M−H]^−^: 478.9420, found: 478.9469.

**Compound 36b:** Yellow solid, final yield 17.8%, m.p. 140.0–141.5 °C; FT-IR (ATR) υ (cm^−1^): 3327, 3149, 3115, 3028, 2975, 1673, 1590, 1415, 1283, 1201, 1008, 786, 635; ^1^H-NMR (600 MHz, DMSO-*d*_6_) *δ*: 1.24 (d, *J* = 10.2 Hz, 6H, imidazole-2’’-CH_3_), 3.36 (m, 1H, imidazole-2’’-CHMe_2_), 5.61 (s, 2H, Ar-2-CH_2_-), 6.94 (s, 1H, Ar-3’-H), 7.07 (d, *J* = 12.6 Hz, 1H, Ar-3-H), 7.10 (s, 1H, Ar-6’-H), 7.53 (s, 1H, imidazole-5’’-H), 7.55 (s, 1H, imidazole-4’’-H), 7.72 (s, 1H, Ar-6-H), 7.85 (d, *J* = 12.6 Hz, 1H, Ar-4-H), 9.75 (brs, 1H, Ar-4’-OH), 10.42 (brs, 1H, Ar-5’-OH); ^13^C-NMR (150 MHz, DMSO-*d*_6_) 20.8×2, 25.2, 48.5, 110.5, 119.3, 119.5, 120.9, 121.9, 122.9, 128.8, 131.5, 133.6, 134.1, 135.6, 139.2, 145.3, 150.9, 152.3, 194.5; ESI-MS *m*/*z*(%) 492.72, 494.71, 496.60 ([M + H]^+^, 48, 100, 58); HR-MS (ESI) calcd for C_20_H_18_Br_2_N_2_O_3_ [M−H]^–^: 492.9580, found: 492.9537.

**Compound 37b:** White solid, final yield 18.3%, m.p. 283.0–285.0 °C; FT-IR (ATR) υ (cm^−1^): 3074, 2985, 2887, 2780, 1664, 1586, 1504, 1408, 1279, 1229, 1151, 1013, 813, 623; ^1^H-NMR (600 MHz, DMSO-*d*_6_) *δ*: 1.19 (t, *J* = 11.4 Hz, 3H, imidazole-2’’-CH_3_), 2.20 (s, 3H, imidazole-4’’-CH_3_), 2.86 (q, *J* = 1.4 Hz, 2H, imidazole-2’’-CH_2_- ), 5.46 (s, 2H, Ar-2-CH_2_-), 6.90 (s, 1H, Ar-3’-H), 7.06 (s, 1H, Ar-6’-H), 7.16 (s, 1H, imidazole-5’’-H), 7.20 (d, *J* = 12.6 Hz, 1H, Ar-3-H), 7.54 (d, *J* = 3.0 Hz, 1H, Ar-6-H), 7.84 (dd, *J* = 3.0, 12.6 Hz, 1H, Ar-4-H), 9.73 (brs, 1H, Ar-4’-OH), 10.43 (brs, 1H, Ar-5’-OH); ^13^C-NMR (150 MHz, DMSO-*d*_6_) 10.0, 11.2, 18.4, 48.1, 110.6, 119.3, 119.7, 121.0, 122.0, 128.5, 128.6, 132.0, 133.3, 133.9, 135.4, 139.6, 145.2, 148.2, 150.9, 194.4; ESI-MS *m*/*z* (%) 491.13, 493.12, 495.14 ([M − H]^−^, 48, 100, 45); HR-MS (ESI) calcd for C_20_H_18_Br_2_N_2_O_3_ [M − H]^−^: 492.9580, found: 492.9518.

**Compound 38b:** White solid, final yield 15.7%, m.p. 210.3–212.0 °C; FT-IR (ATR) υ (cm^−1^): 3415, 3336, 3144, 2927, 2791, 2681, 1654, 1587, 1496, 1416, 1284, 1153, 1012, 700, 635; ^1^H-NMR (600 MHz, DMSO-*d*_6_) *δ*: 5.57 (s, 2H, Ar-2-CH_2_-), 6.83 (s, 1H, Ar-3’-H), 7.01 (s, 1H, Ar-6’-H), 7.10 (d, *J* = 12.6 Hz, 1H, Ar-3-H), 7.49 (d, *J* = 3.0 Hz, 1H, Ar-6-H), 7.59-7.65 (m, 5H, imidazole-Ar-H), 7.76 (d, *J* = 3.0 Hz, 1H, imidazole-5’’-H), 7.79 (dd, *J* = 3.0, 12.6 Hz, 1H, Ar-4-H), 7.89 (d, *J* = 3.0 Hz, 1H, imidazole-4’’-H), 9.70 (brs, 1H, Ar-4’-OH), 10.39 (brs, 1H, Ar-5’-OH); ^13^C-NMR (150 MHz, DMSO-*d*_6_) 49.5, 110.4, 119.3, 120.6, 120.8, 121.9, 123.1, 124.3, 128.8, 129.8 × 2, 130.0 × 2, 131.4, 132.6, 133.6, 134.1, 135.5, 139.0, 145.2, 145.3, 150.8, 194.4; ESI-MS *m*/*z* (%) 525.13, 527.13, 529.13 ([M − H]^−^, 50, 100, 48); HR-MS (ESI) calcd for C_23_H_16_Br_2_N_2_O_3_ [M − H]^−^: 526.9403, found: 526.9444.

**Compound 39b:** Yellow solid, final yield 19.1%, m.p. 190.0–192.0 °C; ^1^H-NMR (600 MHz, DMSO-*d*_6_) *δ*: 2.18 (s, 3H, imidazole-4’’-CH_3_), 2.49 (s, 3H, imidazole-2’’-CH_3_), 5.43 (s, 2H, Ar-2-CH_2_-), 6.90 (s, 1H, imidazole-5’’-H), 7.06 (s, 1H, Ar-3’-H), 7.14 (d, *J* = 13.2 Hz, 1H, Ar-3-H), 7.22 (s, 1H, Ar-6’-H), 7.54 (d, *J* = 3.0 Hz, 1H, Ar-6-H), 7.85 (dd, *J* = 3.0, 12.6 Hz, 1H, Ar-4-H), 9.72 (brs, 1H, Ar-4’-OH), 10.41 (brs, 1H, Ar-5’-OH); ^13^C-NMR (150 MHz, DMSO-*d*_6_) 9.9, 11.0, 48.2, 110.6, 119.3, 119.7, 121.0, 122.0, 128.2, 128.5, 132.2, 133.3, 133.7, 135.3, 139.7, 144.1, 145.2, 150.9, 194.5; ESI-MS *m*/*z* (%) 478.92, 480.90, 482.75 ([M + H]^+^, 50, 100, 60).

**Compound 40b:** White solid, final yield 23%, m.p. 162.0–163.0 °C; ^1^H-NMR (600 MHz, DMSO-*d*_6_) *δ*: 5.88 (s, 2H, Ar-2-CH_2_-), 6.93 (s, 1H, Ar-3’-H), 7.03 (s, 1H, Ar-6’-H), 7.32 (d, *J* = 12.6 Hz, 1H, Ar-3-H), 7.55 (s, 1H, Ar-6-H), 7.59 (t, *J* = 10.8 Hz, 2H, imidazole-Ar-H), 7.72 (d, *J* = 10.8 Hz, 1H, imidazole-Ar-H), 7.81 (dd, *J* = 3.0, 12.6 Hz, 1H, Ar-4-H), 7.87 (d, *J* = 10.8 Hz, 1H, imidazole-Ar-H), 9.47 (s, 1H, imidazole-2’-H), 9.72 (brs, 1H, Ar-4’-OH), 10.38 (s, 1H, Ar-5’-OH); ^13^C-NMR (150 MHz, DMSO-*d*_6_) 48.1, 110.4, 113.8, 115.8, 119.5, 120.8, 122.1, 126.5, 126.8, 128.8, 131.6, 132.3, 132.4, 133.4, 133.6, 135.5, 139.6, 143.2, 145.3, 150.9, 194.7; ESI-MS *m*/*z*(%) 500.88, 502.89, 504.73 ([M + H]^+^, 50, 100, 60).

### 3.2. Biological Assay

EA.hy926 cells were purchased from the Cell Bank of the Chinese Academy of Sciences (Shanghai, China) and maintained in high-glucose DMEM supplemented with 10% fetal bovineserum (FBS; Hyclone, Beijing, China), L-glutamine (2 mM), 100 units/mL of penicillin, and 100 μg/mL streptomycin. Cells were incubated in a humidified incubator aerated with 5% CO_2_ at 37 °C. For all experiments, EA. hy926 cells were cultured in a 96-well plate at a density of 1 × 10^4^/mL, grown to 70–80% confluence, pretreated with designated concentrations 0.3125, 0.625, 1.25, 2.5, 5 and 10 μM of compounds for 4 h, and then exposed to 200 μM H_2_O_2_ (BHKT Clinical Reagent, Beijing, China) for another 6 h in fresh medium. No H_2_O_2_-treated cells were used as controls and were incubated under the same conditions. Cell viability was determined by mitochondrial function using MTT (Sigma Aldrich, St. Louis, MO, USA) testing. The absorbance was detected at 490 nm [11,28].

### 3.3. Molecular Docking

Docking technology is applied to the drug discovery process for predicting the binding mode of a known active ligand. The three-dimensional structures of the compounds were drawn and all molecular modeling calculations were performed in Sybyl 2.0 software (Tripos Associates, St. Louis, MO, USA). All molecule charges were calculated by the Gasteiger-Huckel method. The energy minimization and conformational search were performed using the Tripos force field by Powell method. The X-ray crystal structure of Keap1 protein with ligand IQK701 (PDB code: 4iqk) was retrieved from the RCSB Protein Data Bank [29]. The protein was prepared for docking simulation by extracting original ligand IQK701, deleting all water molecules, adding hydrogen atoms, selecting minimized biopolymer hydrogens, minimizing sidechains, minimizing biopolymer hydrogens without C-Alpha in the Stage Minimization module, and assigning AMBER charge and AMBER7 FF99 force field. Surflex docking module was used. A small molecule ligand was docked in the active site of the binding pocket.

## 4. Conclusions

In summary, a series of novel bromophenols with nitrogen-containing heterocycles piperidine, piperazine, and imidazole were prepared and evaluated for their cytoprotective activity against H_2_O_2_ induced injury in EA.hy926 cells. Most compounds showed moderate-to-potent activity with EC_50_ values in the range of 0.9–7.4 μM. Moreover, the target compound **22b**, a piperazine bromophenol, may be the most promising pharmacological candidate for further cardiovascular drug development owing to its strong cytoprotective ability. Combining with our former studies, the targeting Keap1-Nrf2 protein-protein interaction may be an emerging strategy for this series of halophenols to selectively and effectively activate Nrf2 triggering downstream protective genes that defend against injury.

## Figures and Tables

**Table 1:**
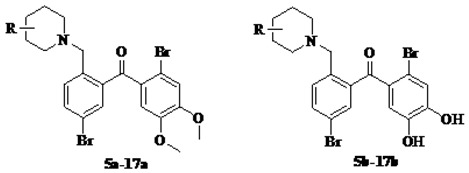
Structures of piperidine compounds **5a–17a**, **5b–17b** and their cytoprotective activity against H_2_O_2_ induced injury in EA.hy926 cells.

R	Compd.	EC_50_^a^ (μM)	Compd.	EC_50_^a^ (μM)
H	**5a**	2.5	**5b**	>30
2-CH_3_	**6a**	>30	**6b**	>30
3-CH_3_	**7a**	>30	**7b**	>30
4-CH_3_	**8a**	>30	**8b**	>30
3-CH_3_, 5-CH_3_	**9a**	>30	**9b**	>30
4-COOH	**10a**	1.9	**10b**	>30
3-COOCH_2_CH_3_	**11a**	>30	**11b**	1.4
4-OH	**12a**	>30	**12b**	4.3
2-CH_3_, 6-CH_3_	**13a**	>30	**13b**	5.4
2,2,6,6-CH_3_	**14a**	1.9	**14b**	6.1
2-CH_2_OH	**15a**	1.7	**15b**	>30
4-CH_2_CH_2_OH	**16a**	>30	**16b**	3.6
4-CH_2_OH	**17a**	5.2	**17b**	>30
	**LM49** ^b^	0.7	Quercetin ^c^	18.0

^a^ EC_50_ values were an average of three separate determinations. ^b^ Used as a lead compound. ^c^ Used as a positive control.

**Table 2:**
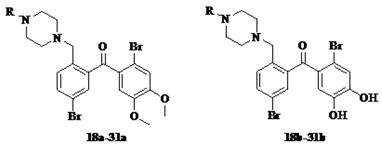
Structures of piperazine compounds **18a**–**31a**, **18b**–**31b** and their cytoprotective activity against H_2_O_2_ induced injury in EA.hy926 cells.

R	Compd.	EC_50_^a^ (μM)	Compd.	EC_50_^a^ (μM)
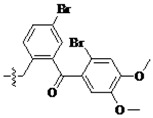	**18a**	>30	**18b**	>30
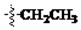	**19a**	>30	**19b**	>30
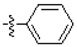	**20a**	>30	**20b**	>30
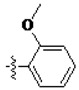	**21a**	3.4	**21b**	3.1
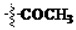	**22a**	>30	**22b**	0.9
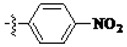	**23a**	>30	**23b**	>30
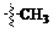	**24a**	7.4	**24b**	6.3
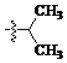	**25a**	>30	**25b**	2.2
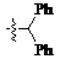	**26a**	>30	**26b**	1.5
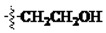	**27a**	2.2	**27b**	>30
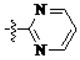	**28a**	3.6	**28b**	>30
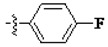	**29a**	4.0	**29b**	>30
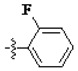	**30a**	4.2	**30b**	>30
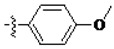	**31a**	4.5	**31b**	>30
	**LM49** ^b^	0.7	Quercetin ^c^	18.0

^a^ EC_50_ values were an average of three separate determinations. ^b^ Used as a lead compound. ^c^ Used as a positive control.

**Table 3:**
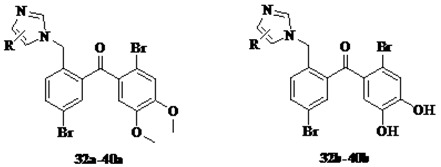
Structures of imidazole compounds **32a**–**40a**, **32b**–**40b** and their cytoprotective activity against H_2_O_2_ induced injury in EA.hy926 cells.

R	Compd.	EC_50_^a^ (μM)	Compd.	EC_50_^a^ (μM)
H	**32a**	1.9	**32b**	>30
2-CH_3_	**33a**	>30	**33b**	>30
4-CH_3_	**34a**	>30	**34b**	>30
2-CH_2_CH_3_	**35a**	>30	**35b**	1.6
2-CH(CH_3_)_2_	**36a**	>30	**36b**	1.3
2-CH_2_CH_3_,4-CH_3_	**37a**	>30	**37b**	1.6
2-Ph	**38a**	>30	**38b**	1.6
2,4-CH_3_	**39a**	4.0	**39b**	>30
[4,5-*d*]Ph	**40a**	3.2	**40b**	>30
	**LM49** ^b^	0.7	Quercetin ^c^	18.0

^a^ EC_50_ values were an average of three separate determinations. ^b^ Used as a lead compound. ^c^ Used as a positive control.
